# A Novel Primary Cilium‐Mediated Mechanism Through which Osteocytes Regulate Metastatic Behavior of Both Breast and Prostate Cancer Cells

**DOI:** 10.1002/advs.202305842

**Published:** 2023-11-15

**Authors:** Stefaan W. Verbruggen, Joanne Nolan, Michael P. Duffy, Oliver M.T. Pearce, Christopher R. Jacobs, Martin M. Knight

**Affiliations:** ^1^ Department of Biomedical Engineering Columbia University in the City of New York New York NY 10027 USA; ^2^ Centre for Bioengineering School of Engineering and Materials Science Queen Mary University of London London E1 4NS UK; ^3^ Department of Mechanical Engineering and INSIGNEO Institute for in silico Medicine University of Sheffield Sheffield S1 3JD UK; ^4^ Centre for Predictive in vitro Models Queen Mary University of London London E1 4NS UK; ^5^ Barts Cancer Institute School of Medicine and Dentistry Queen Mary University of London London EC1M 6AU UK; ^6^ Department of Orthopaedic Surgery Perelman School of Medicine University of Pennsylvania Philadelphia PA 19104 USA

**Keywords:** bone metastatic disease, breast cancer, osteocyte, primary cilium, prostate tumor, TGF‐β, TNF‐α, organ‐on‐a‐chip

## Abstract

Bone metastases are a common cause of suffering in breast and prostate cancer patients, however, the interaction between bone cells and cancer cells is poorly understood. Using a series of co‐culture, conditioned media, human cancer spheroid, and organ‐on‐a‐chip experiments, this study reveals that osteocytes suppress cancer cell proliferation and increase migration via tumor necrosis factor alpha (TNF‐α) secretion. This action is regulated by osteocyte primary cilia and associated intraflagellar transport protein 88 (IFT88). Furthermore, it shows that cancer cells block this mechanism by secreting transforming growth factor beta (TGF‐β), which disrupts osteocyte cilia and IFT88 gene expression. This bi‐directional crosstalk signaling between osteocytes and cancer cells is common to both breast and prostate cancer. This study also proposes that osteocyte inhibition of cancer cell proliferation decreases as cancer cells increase, producing more TGF‐β. Hence, a positive feedback loop develops accelerating metastatic tumor growth. These findings demonstrate the importance of cancer cell‐osteocyte signaling in regulating breast and prostate bone metastases and support the development of therapies targeting this pathway.

## Graphical abstract

This study identifies a previously unknown molecular mechanism common to both breast and prostate cancer cells, whereby they hijack bone cells to stimulate growth of bone metastases. This cell crosstalk is investigated, finding that osteocytes inherently suppress cancer cell proliferation. This is regulated by the osteocyte's primary cilium, which cancer cells can knock down to stimulate bone metastases.

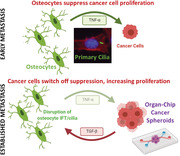

## 1 Introduction

Breast and prostate cancers are the two most prevalent types worldwide with over 1 million deaths globally each year, predominantly due to metastatic disease.^[^ ^1^ ^]^ Tumor metastasis is one of the most critical events in cancer development, after which five‐year patient survival rates in the UK decreases from 90–98% when diagnosed at Stage I‐II, to 26% at Stage IV for breast cancer, and 99% to 30% at equivalent stages for prostate cancer.^[^ ^2^ ^]^ One of the most common sites for tumor metastasis is bone, with over 450,000 patients currently suffering from this condition in the US.^[^ ^3^ ^]^ Indeed, it is the preferred site for breast and prostate cancer metastasis,^[^ ^4^ ^]^ with 65−75% of patients with metastases developing skeletal lesions that account for >80% of all cases of metastatic bone disease.^[^ ^5^, ^6^ ^]^ Recent research has shown that metastatic spread occurs early in breast cancer development,^[^ ^7^, ^8^ ^]^ and disseminated tumor cells are present in bone marrow by the time primary tumors are diagnosed.^[^ ^9^ ^]^ Once metastatic tumors develop in bone, the median survival time is 1–4 years,^[^ ^5^ ^]^ indicating that metastasis is now a common cause of death and suffering in breast and prostate cancer patients.^[^ ^10^ ^]^ Despite this, the mechanisms by which metastatic tumors develop in bone from disseminated tumor cells, and the interactions between bone cells and cancer cells, remain poorly understood.

Much research into the effects of metastatic cancer cells in bone has focussed on the marrow, and interactions with the array of bone cell types found there.^[^ ^10^ ^]^ However, osteocytes represent >90% of bone cells,^[^ ^11^ ^]^ are spread throughout mineralized bone tissue and are known to be the primary regulator of this environment, orchestrating the behavior of other bone cells in response to mechanical loading.^[^ ^12^ ^]^ Despite their important regulatory role, osteocyte interactions with cancer cells have only recently begun to be explored. Initial conditioned media experiments showed that signals secreted by osteocytes could alter proliferation and migration in a range of breast and prostate cancer cells,^[^ ^13^ ^]^ Their importance has been further demonstrated in breast cancer through the application of mechanical loading, showing that conditioned media from osteocytes stimulated using oscillatory fluid flow can reduce the trans‐endothelial migration of triple‐negative MDA‐MB‐231 breast cancer cells, possibly through signaling to osteoclasts and endothelial cells as intermediaries.^[^ ^14^, ^15^ ^]^ Additional research into oestrogen receptor‐positive MCF‐7 breast cancer cells observed increased proliferation and migration when treated with conditioned media of mechanically stimulated osteocytes, identifying CXCL1/2 as a potential mechanism.^[^ ^16^ ^]^ A significant recent advance in the field of cancer research is the development of microfluidic platforms^[^ ^17^ ^]^ designed to replicate extravasation of MDA‐MB‐231 cells in the presence of osteocytes, demonstrated reduced extravasation with mechanical stimulation of the bone cells.^[^ ^18^ ^]^ In a similar manner, our group has recently observed increased invasive behavior in both breast and prostate cancer cells when osteocytes were mechanically stimulated in an organ‐chip model of metastatic bone disease.^[^ ^19^ ^]^ However, almost no research has investigated the cytokine crosstalk between cancer cells and osteocytes in co‐culture, which is perhaps more representative of an established metastatic tumor microenvironment.

It is clear that osteocytes are emerging as a key regulator of metastasis in breast cancer, and possibly also in prostate cancer. However, the molecular mechanisms through which osteocytes regulate cancer cells remain unknown. Even less is known about how a developing mass of tumor cells affects osteocytes, with possible consequences for modulation of cancer cell behavior and downstream bone remodeling. Therefore, the objective of this study is to take a step‐wise approach, selectively adjusting conditioned media and co‐culture studies to replicate osteocyte‐ and cancer‐dominated environments found in vivo, teasing apart the mechanisms through which breast and prostate cancer cells interact with bone cells and the bone microenvironment to form metastatic tumors.

## 2 Results

### 2.1 Osteocyte Conditioned Media, but Not Co‐Culture, Suppresses Proliferation and Increases Migration in Both Breast and Prostate Cancer Cells

**Figure 1:**
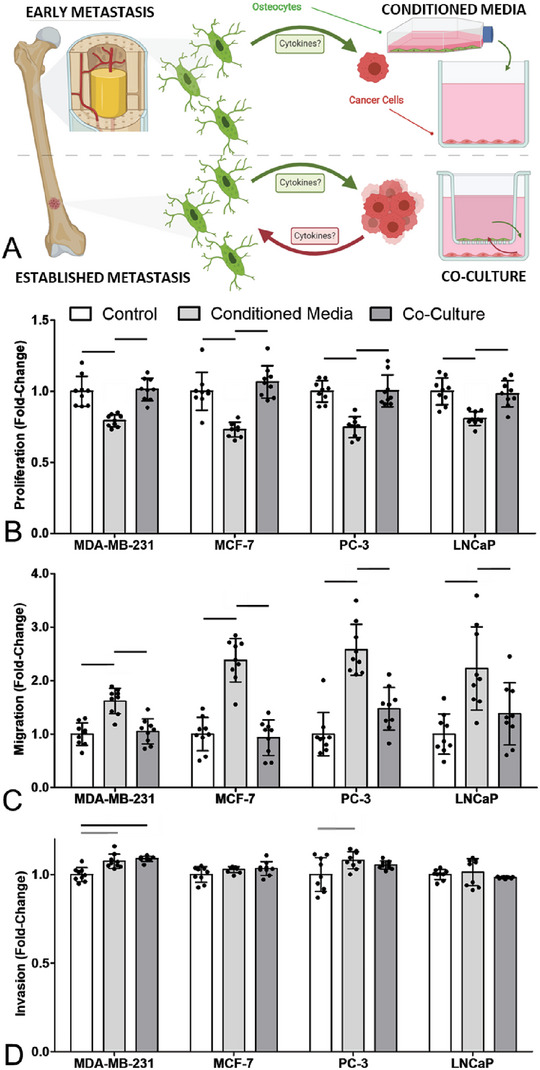
Osteocyte conditioned media inhibited proliferation and increased migration of breast and prostate cancer cells, with absence of this effect in co‐culture suggesting the existence of a feedback mechanism in vivo. A) Schematic of conditioned media model of early metastasis, and co‐culture model of late metastasis. Fold‐change in B) proliferation, C) migration and D) invasion of breast (MDA‐MD‐231 & MCF‐7) and prostate (PC‐3 & LNCaP) cancer cell lines, after 48 h in conditioned media or co‐culture with the MLO‐Y4 osteocyte‐like cell line (*n* = 9). Bar charts represent mean ± standard deviation. Statistically significant differences indicated by horizontal lines based on one‐way ANOVA with Bonferroni post‐hoc test (light gray *p* < 0.05, dark gray *p* < 0.01, black *p* < 0.001).

We first examined the effect of osteocyte conditioned media (CM) on cancer cell behavior and compared this with the effect of osteocytes in co‐culture (Co‐C) with the cancer cells. We suggest that the conditioned media experiments are more representative of early metastasis where there are insufficient cancer cell numbers to regulate the osteocytes, while co‐culture maybe more representative of established metastatic colonies (see schematic in **Figure** **1**A). Addition of conditioned media from osteocytes resulted in significantly reduced proliferation, of up to 26%, in both of the breast cancer cell lines and both of the prostate cancer cell lines (Figure 1B). In contrast, conditioned media resulted in large increases in migration (up to 144%) in each cancer cell type (Figure 1C). Hence, secreted factors from osteocytes push cancer cells into a more migratory, anti‐proliferative phenotype. While a small, but significant, increase in invasion of MDA‐MB‐231 breast cancer cells was observed with conditioned media and co‐culture, no significant changes were observed in other cell lines with regard to invasion (Figure 1D). This pattern continued in other experiments and thus the remaining invasion results have been included in Figure S1 (Supporting Information)

In contrast, co‐culture with osteocyte cells made no significant difference to either proliferation or migration behavior of cancer cells, when compared to control cancer cells in standard media. This indicates the osteocyte regulation of cancer cells, as seen with conditioned media, is blocked by the cancer cells, suggesting changes in crosstalk between the two cell populations as metastatic colonies develop.

### 2.2 Osteocyte Regulation of Breast and Prostate Cancer Cell Proliferation and Migration is Inhibited by TGF‐β Released from Cancer Cells

**Figure 2:**
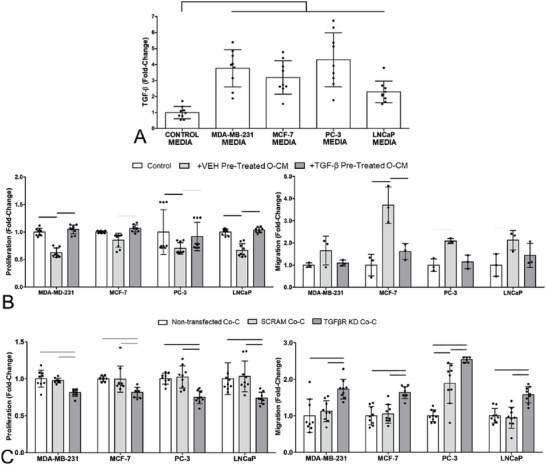
TGF‐β, secreted by breast and prostate cancer cells, blocks the disruption of cancer cells via osteocyte conditioned media. Similarly, knockdown of osteocyte TGF‐β receptor I in co‐culture resulted in similar effects to normal osteocyte conditioned media, inhibiting proliferation and increasing migration of breast and prostate cancer cells, indicating TGF‐β plays a role in feedback loop. A) Fold‐increases in TGF‐β secretion by cancer cell lines compared to standard culture media, as measured by ELISA. Fold‐change in B) proliferation and migration of breast (MDA‐MD‐231 & MCF‐7) and prostate (PC‐3 & LNCaP) cancer cell lines, after 48 h in osteocyte CM, with or without TGF‐β pre‐treatment (*n* = 9). Data normalized to untreated control cancer cells. Quantification of C) proliferation and migration of breast and prostate cancer cell lines, after 48 h in co‐culture (Co‐C) with osteocytes in which TGF‐β receptor I has been knocked down with siRNA (TGFβR KD) (*n* = 9). Data normalized to co‐culture with non‐transfected osteocytes and shown alongside scrambled controls (SCRAM). Bar charts represent mean ± standard deviation. Statistically significant differences indicated by horizontal lines based on one‐way ANOVA with Bonferroni post‐hoc test (light gray *p* < 0.05, dark gray *p* < 0.01, black *p* < 0.001).

The observation that osteocyte regulation of cancer cells is absent in co‐culture suggests soluble factors from cancer cells may be responsible for suppressing this osteocyte behavior. A prime candidate for mediating this crosstalk was transforming growth factor (TGF‐β), known to be secreted by many cancer cell types. Indeed, we found significant amounts of TGF‐β were released by all four cancer cell lines tested here, with 2.4 to 4.3 fold increases compared to standard control media (**Figure** **2**A, with concentrations in Figure S2, Supporting Information). To test the involvement of TGF‐β in regulating this behavior, we pre‐treated osteocytes with TGF‐β and then collected conditioned media, with this treatment inhibiting the decreased proliferation and increased migration stimulated by osteocyte conditioned media (Figure 2B). To further test this, a knockdown of TGF‐β receptor I in osteocytes in co‐culture was performed via siRNA transfection (confirmation of knockdown in Figure S3, Supporting Information). Osteocyte TGF‐β receptor I knockdown in co‐culture resulted in significantly decreased cancer cell proliferation (by up to 24%, Figure 2C) and significantly increased cancer cell migration (by up to 149%, Figure 2C; Figure S3 Supporting Information), when compared to both non‐transfected and scrambled transfected controls. The behavior of cancer cells in co‐culture with osteocytes lacking TGF‐β receptor I, is strikingly similar to that observed in conditioned media in Figure 1. Together, these results indicate that cancer cell secretion of TGF‐β blocks the osteocyte regulation of cancer cell proliferation and migration.

### 2.3 TGF‐β Secreted by Breast and Prostate Cancer Cells Reduces Expression of Osteocyte Primary Cilia and IFT88

**Figure 3:**
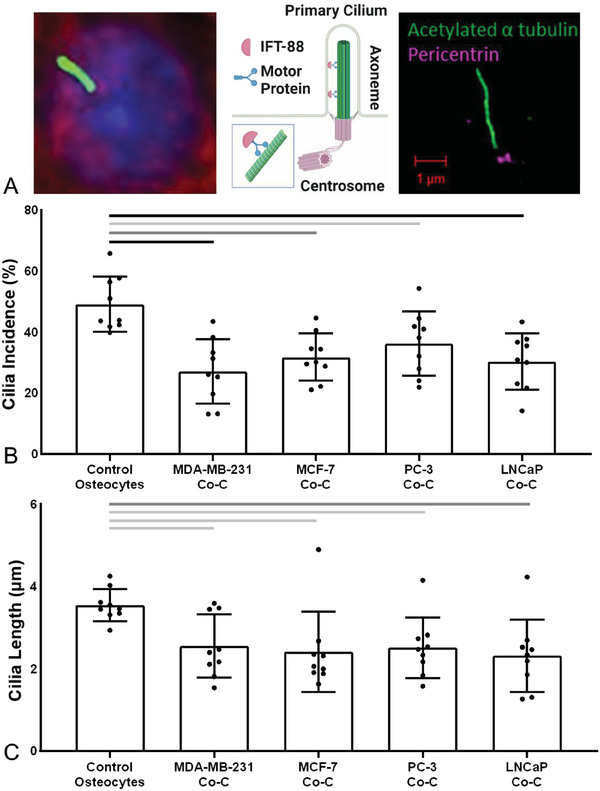
Osteocyte primary cilia expression was reduced in co‐culture with breast and prostate cancer cells. A) Schematic of the osteocyte primary cilium demonstrating how IFT88 binds to motor proteins to construct the axoneme. Imaging performed using confocal microscopy (nuclei = blue, DAPI; F‐actin cytoskeleton = red, phalloidin; axoneme = green, acetylated α‐tubulin) and super‐resolution Structured Illumination Microscopy (SR‐SIM) (centrosome/basal body = magenta, pericentrin; axoneme = green, acetylated α‐tubulin). B,C) Primary cilia expression in osteocytes (control) and the effect of co‐culture with each breast and prostate cancer cell line (Co‐C). Quantification of B) cilia incidence and C) cilia length. Bar charts represent mean ± standard deviation for *n* = 9 technical replicates. Statistically significant differences indicated by horizontal lines based on one‐way ANOVA with Bonferroni post‐hoc test (light gray *p* < 0.05, dark gray *p* < 0.01, black *p* < 0.001). Data points for each replicate indicate the incidence based >200 cells and median values for cilia length based on 100 cilia.

**Figure 4:**
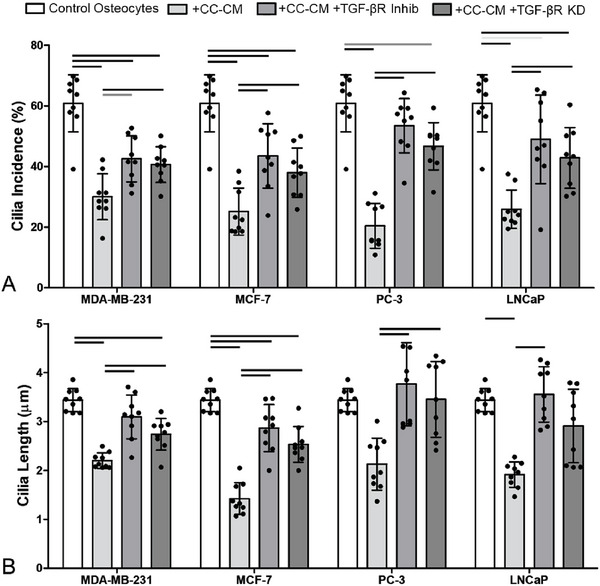
Conditioned media from breast and prostate cancer cells reduced osteocyte primary cilia expression. This effect was inhibited by disruption of TGF‐β receptor I via small molecule inhibitor or siRNA. (A‐B) Primary cilia expression in osteocytes (control) and the effect of cancer cell conditioned media (CC‐CM) from cancer cells. Conditioned media was applied either on its own (+CC‐CM) or after pre‐treatment with TGF‐β receptor I small‐molecule inhibitor (+CC‐CM +TGF‐βR Inhib) or following siRNA knockdown of TGF‐β receptor I (+CM +TGF‐βR KD). Quantification of A) cilia incidence and B) cilia length. Bar charts represent mean ± standard deviation for *n* = 9 technical replicates. Statistically significant differences indicated by horizontal lines based on one‐way ANOVA with Bonferroni post‐hoc test (light gray *p* < 0.05, dark gray *p* < 0.01, black *p* < 0.001). Data points for each replicate indicate the incidence based >200 cells and median values for cilia length based on >100 cilia.

**Figure 5:**
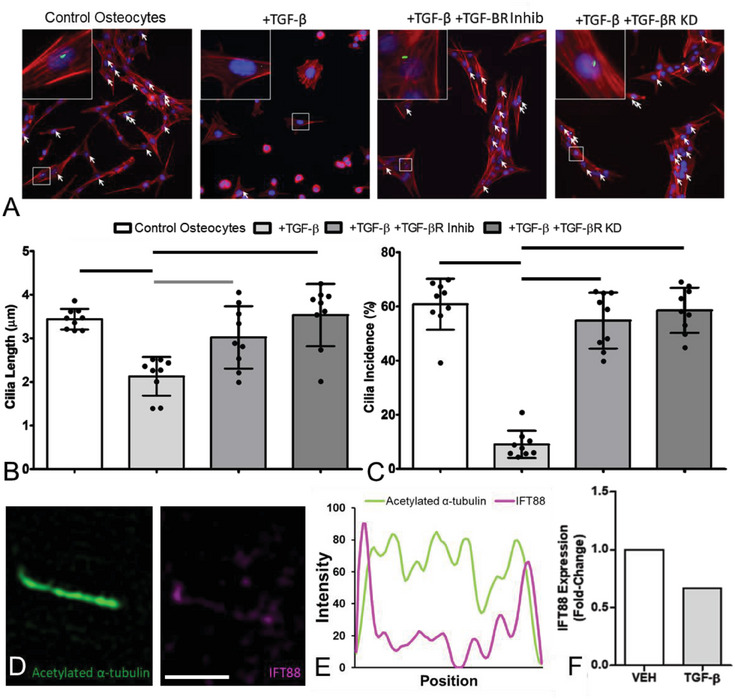
TGF‐β, released by breast and prostate cancer cells, significantly reduced expression of osteocyte primary cilia and IFT88. A) Immunofluorescent images of osteocytes (nuclei = blue, DAPI; primary cilia = green, acetylated α‐tubulin; F‐actin cytoskeleton = red, phalloidin), showing shorter and fewer primary cilia (indicated by white arrows) with addition of TGF‐β. B,C) This effect was reversed by either the addition of a TGF‐β receptor I small‐molecule inhibitor (TGF‐βR Inhib) or knockdown of TGF‐β receptor I via siRNA transfection (TGF‐βR KD) (*n* = 9). D) Super‐resolution Structured Illumination Microscopy (SR‐SIM) images of IFT88 present in an osteocyte cilium, showing E) the intensity profile along the axoneme (IFT88 = magenta; axoneme = green, acetylated α‐tubulin). F) TGF‐β treatment of osteocytes also decreased expression of IFT88 (*n* = 1) as measured by qPCR, when compared to vehicle‐treated controls. Bar charts represent mean ± standard deviation. Statistically significant differences indicated by horizontal lines based on one‐way ANOVA with Bonferroni post‐hoc test (light gray *p* < 0.05, dark gray *p* < 0.01, black *p* < 0.001).

We next determined the effect of cancer cell‐secreted TGF‐β on the osteocytes as a first step to identifying the mechanism through which osteocytes regulate cancer cells. The primary cilium, a slender organelle typically protruding from the cell surface (shown via confocal and SR‐SIM images in **Figure** **3**A), is a key chemosignaling nexus present in almost all mammalian cells, with the notable exception of proliferating cancer cells where it is instead associated with increased drug resistance.^[^ ^20^ ^]^ In osteocytes, it is known to govern a range of important pathways, including Wnt, Hedgehog and mechanosignaling.^[^ ^21^ ^]^ In standard cell culture, 40–60% of osteocytes expressed a primary cilium, with lengths of ≈3–4 µm (**Figure** **4**). Immunofluorescence imaging of osteocytes demonstrated that TGF‐β reduced osteocyte primary cilia prevalence and length, in agreement with findings reported in other cell types.^[^ ^22^, ^23^, ^24^ ^]^ Furthermore, conditioned media from each cancer cell line also induced similar changes in cilia expression with shorter cilia and prevalence decreased to 20% (Figure 4; Figure S4, Supporting Information). Similarly, cancer cells also down‐regulated cilia expression in osteogenically‐differentiated human MSCs (Figure S5, Supporting Information). Cancer cell conditioned media also induced decreased mechanosensitivity in osteocytes as measured by cyclooxygenase‐2 (COX‐2) mRNA expression, an important regulator of downstream osteogenic signaling (Figure S6A, Supporting Information). Using siRNA for intraflagellar transport protein 88 (IFT88) to knockdown osteocyte primary cilia, we found that this disrupted osteocyte mechanosensitivity did indeed abrogate the mechano‐regulated control of breast cancer cell proliferation, as we observed previously.^[^ ^19^ ^]^ However, no effect on prostate cancer cells was observed (Figure S6B, Supporting Information). Reduction in cilia expression caused by TGF‐β or cancer cell conditioned media was also associated with rounding of osteocytes, as measured by significant changes in circularity and cell area (Figure 4; Figures S4 and S7, Supporting Information). These changes in morphology suggest reduced actin tension, which has previously been shown to inhibit cilia expression.^[^ ^25^ ^]^ TGF‐β treatment also reduced osteocyte expression of the intraflagellar transport gene, IFT88 (**Figure** **5**), which controls ciliagenesis as demonstrated in various cell types including osteoblasts and chondrocytes.^[^ ^22^, ^23^, ^24^ ^]^ We then sought to block TGF‐β regulation of osteocyte primary cilia expression using either small molecule inhibitor of TGF‐β receptor I or siRNA transfection. The effectiveness of both approaches was confirmed by the complete inhibition of any changes in cilia length or prevalence induced by TGF‐β treatment (Figure 5). Disruption of TGF‐β receptor I was then shown to inhibit the effect of cancer cell conditioned media on osteocyte cilia expression (Figure 5). Thus, we demonstrate that cancer cells suppress the expression of osteocyte primary cilia/IFT88 via the release of TGF‐β.

### 2.4 Osteocyte Primary Cilia/IFT88 are Required for Regulation of Cancer Cell Proliferation and Migration

**Figure 6:**
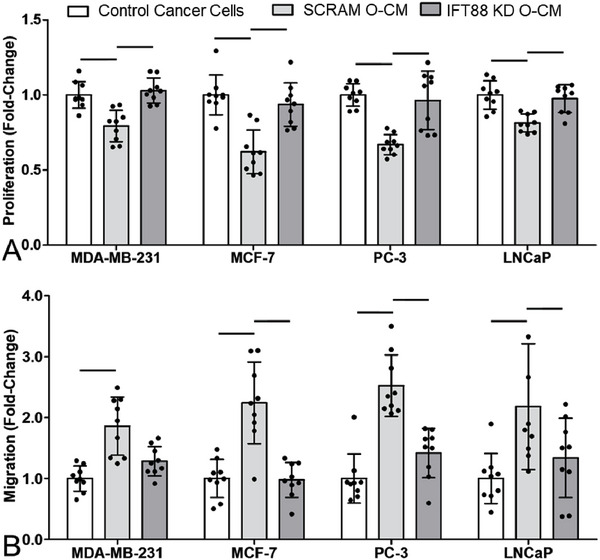
Knockdown of osteocyte primary cilia/IFT88 altered cancer cell behavior via conditioned media to match control and co‐culture conditions. Fold‐change in A) proliferation and B) migration of breast (MDA‐MD‐231 & MCF‐7) and prostate (PC‐3 & LNCaP) cancer cell lines, after 48 h in standard control media, conditioned media from MLO‐Y4s transfected with scrambled (SCRAM O‐CM) or IFT88 (IFT88 KD O‐CM) siRNA (*n* = 9). Bar charts represent mean ± standard deviation. Statistically significant differences indicated by horizontal lines based on one‐way ANOVA with Bonferroni post‐hoc test (light gray *p* < 0.05, dark gray *p* < 0.01, black *p* < 0.001).

We have shown that the disruption of cancer cell proliferation and migration by osteocytes is suppressed by release of TGF‐β from cancer cells, and that TGF‐β also disrupts osteocyte cilia and IFT88 expression (Figures 4 and 5). We, therefore, sought to determine whether the inhibition of osteocyte regulation of cancer cell behavior was due to the reduced expression of osteocyte cilia/IFT88 or via another TGF‐β‐mediated pathway. To achieve this we employed a knockdown of osteocyte primary cilia via IFT88 siRNA transfection as confirmed by confocal immunofluorescence, qPCR, and western blot (Figure S8, Supporting Information). This resulted in reduced cilia expression without any significant changes in cell morphology (Figure S9, Supporting Information). Conditioned media from osteocytes transfected with scrambled siRNA significantly reduced cancer cell proliferation and increased migration compared to that seen in cancer cells alone (**Figure** **6**A,B). This response occurred in the two breast cancer cell lines and in the two prostate cancer cell lines, mirroring the effect of conditioned media from non‐transfected osteocytes (Figure 1). However, this response was blocked when using conditioned media from osteocytes transfected with siRNA to IFT88, with significant differences between the response between IFT88 siRNA and scrambled control (Figure 6A,B). Consequently, there were no statistically significant differences in proliferation or migration compared to cancer cells alone. Thus, these effects of conditioned media from osteocytes with IFT88/cilia knockdown, replicate the behavior seen in co‐culture (Figure 1).

### 2.5 Disruption of Osteocyte Primary Cilia Inhibits TNF‐α Release, which Modulates Cancer Cell Behavior

**Figure 7:**
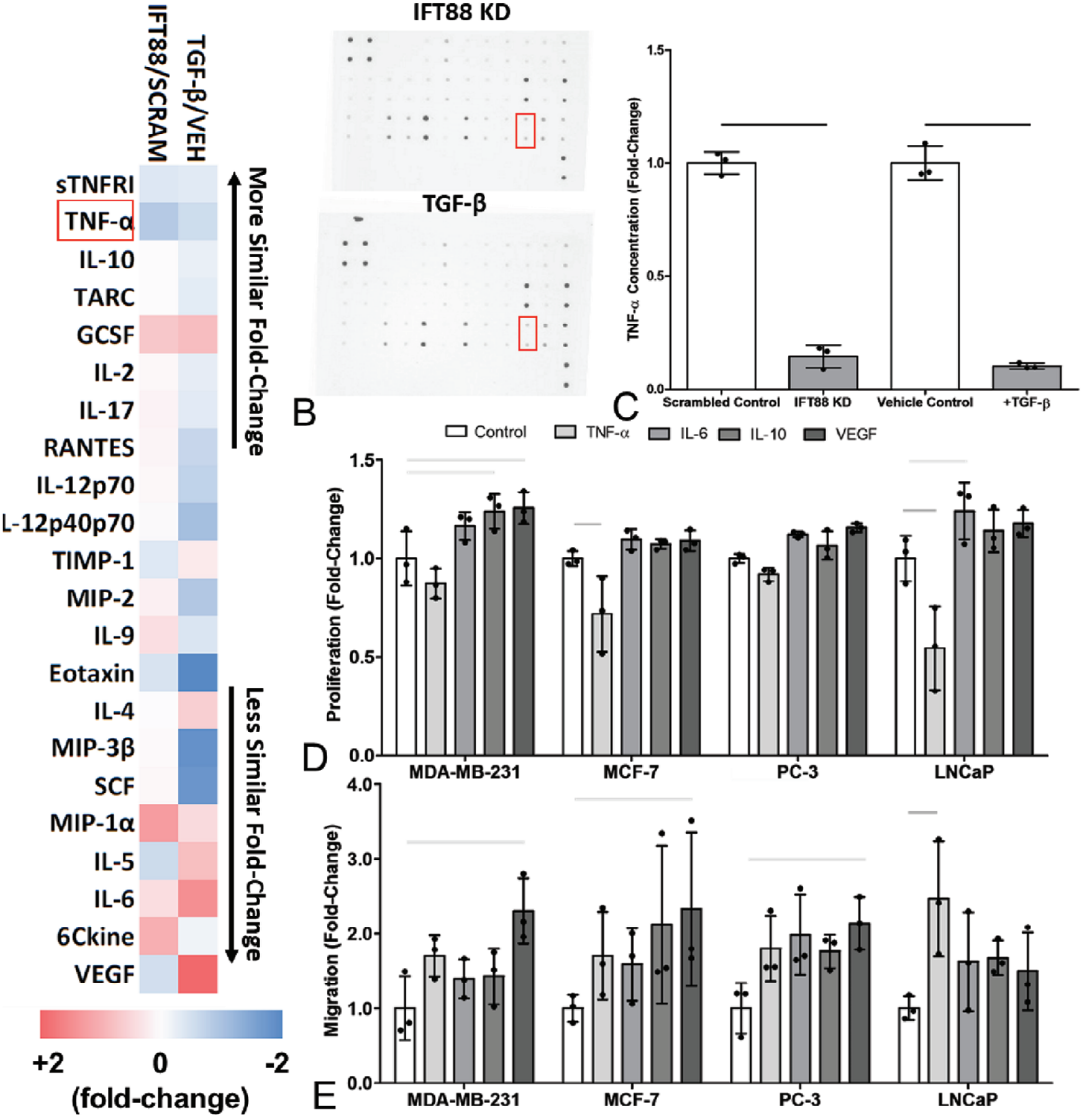
Knockdown of osteocyte primary cilia, via IFT‐88 siRNA or TGF‐β treatment, inhibited production of TNF‐α (highlighted in red), a cytokine that decreased proliferation and increased migration in cancer cells. A,B) Cytokine targets were selected based on significant change relative to control CM in a cytokine array of 32 inflammatory proteins (only those with a greater than 25% change shown), for both the most similar (IL‐10 and TNF‐α) and least similar (IL‐6 and VEGF) expression changes. C) An ELISA confirmed significant decreases in TNF‐α concentration in MLO‐Y4 CM with IFT88 siRNA knockdown or TGF‐β treatment. Fold change in D) proliferation and E) migration in breast (MDA‐MD‐231 & MCF‐7) and prostate (PC‐3 & LNCaP) cancer cell lines, after 24 h treatment with selected inflammatory cytokines (*n* = 3). Bar charts represent mean ± standard deviation. Statistically significant differences indicated by horizontal lines based on one‐way ANOVA with Bonferroni post‐hoc test (light gray *p* < 0.05, dark gray *p* < 0.01, black *p* < 0.001).

We have now shown that osteocytes suppress cancer cell proliferation and increase migration and that this response is blocked by cancer cell secretion of TGF‐β. In this final section, we sought to identify the paracrine signaling mechanism through which osteocytes regulate cancer cell behavior. We have shown that this signaling is blocked by inhibition of osteocyte primary cilia/IFT88 expression via cancer cell secretion of TGF‐β. Therefore, a cytokine array of 32 standard pro‐inflammatory targets was used to compare media from TGF‐β treated and IFT88 KD osteocytes with their respective vehicle or scrambled siRNA controls (**Figure** **7A,B**; Figures S10 and S11, Supporting Information). A protein‐protein interaction (PPI) network of the targets present in the cytokine array was generated using STRING^[^ ^26^ ^]^ indicating a cluster of interacting proteins (Figure S12, Supporting Information), four of which were highly differentially expressed in our cytokine arrays. Interleukin 10 (IL‐10) and tumor necrosis factor alpha (TNF‐α) demonstrated >1.5‐fold changes in secretion that were similar in both TGF‐β treated and IFT88 KD osteocytes as shown in the heatmap in Figure 7A. Subsequent analysis of TNF‐α via an ELISA confirmed this similarity in behavior (Figure 7C; Figure S13, Supporting Information). By contrast, interleukin 6 (IL‐6) and vascular endothelial growth factor (VEGF) demonstrated the least similar responses between the two groups.

**Figure 8:**
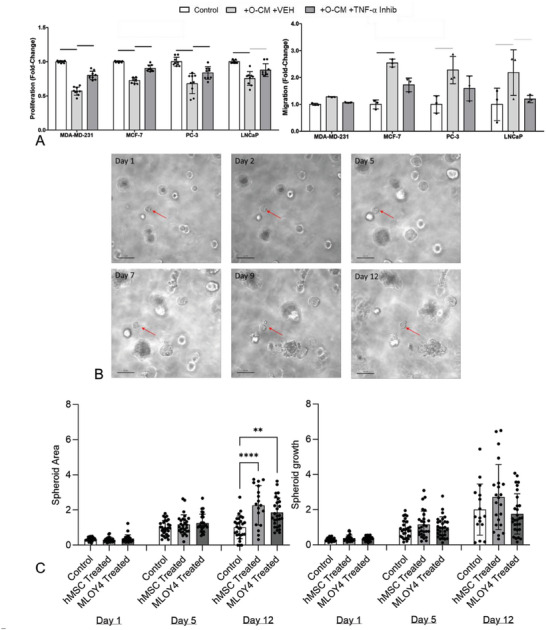
Pre‐treatment of cancer cells with a TNF‐α Inhibitor blocks the effect of osteocyte conditioned media on cancer cells, replicating the effect of co‐culture, and effects measured in monolayer culture are replicated using 3D cancer spheroids. A) Fold‐change in proliferation and migration of breast (MDA‐MD‐231 & MCF‐7) and prostate (PC‐3 & LNCaP) cancer cell lines, after 48 h in osteocyte CM or with pre‐treatment with a TNF‐α small molecule inhibitor (*n* = 9,3). B) 3D spheroids generated using MCF‐7 and PC‐3 cells were monitored over 12 days, finding C) that similar effects on proliferation were measured whether conditioned media was from mouse MLO‐Y4 osteocyte cell line or osteogenically‐differentiated human MSCs. Bar charts represent mean ± standard deviation. Statistically significant differences indicated by horizontal lines based on one‐way ANOVA with Bonferroni post‐hoc test (light gray *p* < 0.05, dark gray *p* < 0.01, black *p* < 0.001) or by asterisks using the same test (** *p* < 0.05, **** *p* < 0.0001).

When these four pro‐inflammatory factors were added to cancer cells, TNF‐α was the only cytokine to cause a decrease in proliferation alongside an increase in migration (Figure 6D,E). The similarity of this pattern of behavior to that induced by osteocyte conditioned media, implicates TNF‐α as a potential cytokine secreted by osteocytes to regulate cancer cell proliferation and migration. This was confirmed via addition of a TNF‐α small‐molecule inhibitor to osteocyte conditioned media, which significantly inhibited the decreased cancer cell proliferation and increased migration such that there were no significant differences compared to cancer cells alone (**Figure** **8**A).

**Figure 9:**
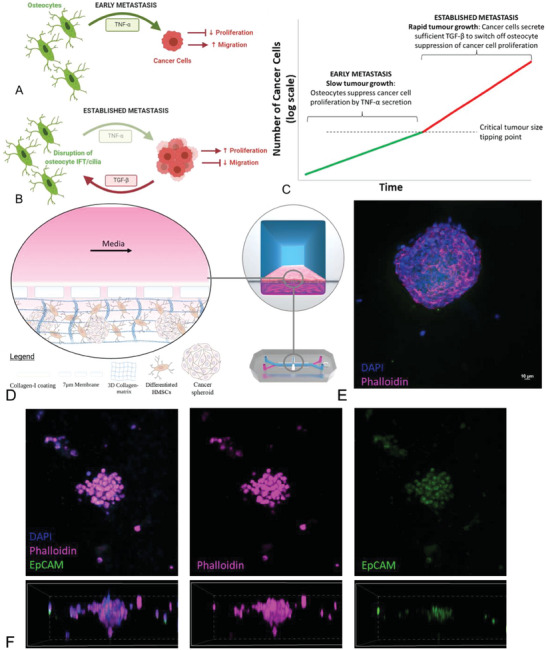
TNF‐α secreted by osteocytes inhibits proliferation and encourages migration in breast and prostate cancer cells, with this inherent anti‐cancer mechanism dependent on IFT88/primary cilium expression. TGF‐β secretion by cancer cells blocks this mechanism, allowing further proliferation of cancer cells and potentially reaching a tipping point beyond which tumor growth accelerates. A) Working model of osteocyte‐cancer cell interactions in early metastasis when few cancer cells are present, followed by a working model of B) a larger tumor in established metastasis, with potential activated/inhibited signaling identified. C) Model of cancer cell proliferation under the influence of osteocyte signaling, with the proposed feedback loop leading to a tipping point after which cell growth accelerates. D) Schematic of a microfluidic organ‐on‐a‐chip model of human breast and prostate cancer metastases developed using the Emulate Inc. platform to further test this mechanism, and E) associated confocal microscopy images showing the human cancer cell spheroids suspended in hydrogel within the organ‐chip. F) Co‐culture of the cancer cell spheroids alongside osteogenically‐differentiated human MSCs, with nuclei immunostained for DAPI (blue), cell cytoskeleton by phalloidin for F‐actin (magenta), and cancer cells are selectively stained with EpCAM and shown as a z‐stack (green), with hMSCs visible in the merged image as cells not expressing EpCAM.

Thus, we suggest the following bi‐direction or feedback mechanism regulating cancer cell behavior as shown schematically in **Figure** **9**:

‐TNF‐α is secreted by osteocytes, which in early metastasis, vastly outnumber cancer cells, suppressing proliferation of breast and prostate cancer cells, while encouraging migration (Figure 9A).
‐This behavior is dependent on osteocyte primary cilia and associated IFT88, which are inhibited in established metastatic colonies by increased TGF‐β secreted by the higher number of cancer cells (Figure 9B).
‐This disruption of the cilia/IFT88 expression blocks TNF‐α secretion from osteocytes, thereby switching off both the inhibition of cancer cell proliferation and the up‐regulation of migration.
‐Hence, increased numbers of cancer cells produce more TGF‐β, further disabling osteocyte TNF‐α secretion in a positive feedback loop reducing cancer cell migration and increasing proliferation, thereby accelerating metastatic tumor growth.

This hypothesis is further corroborated by RNAseq studies publicly available as part of “The Cancer Genome Atlas (TCGA)” and analyzed using KMPlot^[^ ^27^ ^]^ in which there is a trend of lower rates of disease‐free survival in breast and prostate cancer patients with low expression of TNF‐α receptors (Figure S14A, Supporting Information). These data indicate a selective advantage for cancer cells that cannot sense this osteocyte signaling. Similarly, gene transcription data from previous metastatic databases analyzed using TNMPlot^[^ ^28^ ^]^ showed a trend of decreased expression of TNF‐α receptors in metastatic tissue compared to primary breast or prostate tumors (Figure S14B, Supporting Information), matching with our feedback mechanism hypothesis.

This hypothesis is used to develop a simple numerical model of metastatic cancer cell growth as presented in Figure 8C. Taking the average doubling rate of the cancer cells via fold change in proliferation observed over 24 and 48 h periods, and applying an ≈20% suppression of proliferation observed via osteocytes, we have modeled an estimated cell growth curve (Figure 9C). This predicts an initial slow growth rate of cancer cells up to a tipping point, beyond which growth rate accelerates as osteocyte suppression is attenuated by cancer cells.

More complex in vitro models, such as organ‐on‐a‐chip models composed of all‐human cells and incorporating a more complex 3D tumor microenvironment, will strengthen further analysis of this novel mechanism.^[^ ^17^ ^]^ Therefore, we have constructed tumor spheroids from the cancer cell lines and tested the effects of both the mouse osteocyte cell line and osteogenically‐differentiated human MSCs on their proliferation, finding no significant difference in spheroid growth between the mouse and human cells (Figure 8B,C). A trend toward increased proliferation was observed when the MCF‐7 spheroids were stimulated with human MSC conditioned media, which may arise from the fact that, while these cells are osteogenically differentiated they cannot be fully pushed down the differentiation pathway toward terminally differentiated osteocytes. While we have not explored endothelial to mesenchymal transition (EMT) in this study, this process may play a role in some of the changes seen in our model. To further explore these questions, we have now built upon our previous organ‐on‐a‐chip model of cancer cells and osteocytes^[^ ^19^ ^]^ to build an all‐human organ‐chip model of 3D suspended osteogenically‐differentiated human MSCs and 3D spheroids of human cancer cells (Figure 9D,E). Future work with this new bone metastasis organ‐chip model will allow further investigation and therapeutic testing in a more complex 3D human tumor microenvironment.

## 3 Discussion

This study presents a novel cytokine mechanism, common to both breast and prostate cancer, whereby osteocytes can suppress cancer cell proliferation via TNF‐α secretion. This anti‐proliferative mechanism is primary cilium‐ or IFT88‐dependent, and can be suppressed by TGF‐β released by cancer cells. These findings present the intriguing prospect of a positive feedback loop, whereby breast and prostate cancer cells disable this anti‐cancer mechanism to encourage further proliferation of cancer cells, greater production of TGF‐β, and further knockdown of osteocyte regulation. These mechanisms present new therapeutic targets to prevent further growth of bone metastatic tumors, including potential ciliotherapies.

This study used MLO‐Y4 osteocyte‐like cells of mouse origin, with breast (MDA‐MB‐231 and MCF‐7) and prostate (PC‐3 and LNCaP) cancer cells of human origin. Despite the limitations provided by the species difference, these cancer cell lines are very well‐established and have been studied extensively in mouse models of cancer cell metastasis.^[^ ^29^, ^30^ ^]^ A key limitation of the MLO‐Y4 cell line is low sclerostin expression, which prevents us from investigating the effect of this pathway in bone metastasis. However, MLO‐Y4 cells are the most well‐understood osteocyte cell‐line.^[^ ^31^ ^]^ The use of these established cell lines provided greater reproducibility than would be possible with human primary cells, which are terminally differentiated and would have been difficult to obtain in the quantities required for this study. In the absence of terminally differentiated primary human osteocytes, we repeated a number of experiments using osteogenically‐differentiated primary human MSCs, finding no significant difference between our results and those from the MLO‐Y4 cell line. The approach of sequential conditioning and co‐culture is less physiologically representative than an in vivo system, but allowed us to isolate effects and investigate specific molecular interactions between two cell types. Moreover, the use of conditioned media and transwell co‐culture means only soluble factors could have an impact on our observations, and this is more representative than direct contact with osteocytes, which are dispersed and embedded in the bone matrix. Finally, there are well‐accepted limitations of 2D monolayer cultures as being unrepresentative of the tumor microenvironment, and so we have replicated a number of our findings in 3D spheroids of cancer cell lines, and have developed a fully human organ‐on‐a‐chip model to test this mechanism in a more physiologically relevant microenvironment.

Osteocyte regulation of cancer cell behavior has been observed previously, with conditioned media found to alter proliferation, migration, invasion, and extravasation of cancer cells.^[^ ^13^, ^14^, ^15^, ^16^, ^18^, ^19^ ^]^ A recent study has begun the important work of explaining these changes, proposing a potential CXCL1/2‐mediated mechanism through which osteocytes may regulate proliferation of breast cancer cells.^[^ ^16^ ^]^ However, a general mechanism through which osteocytes control both breast and prostate cancer cell behaviors remains unknown. Our experiments demonstrated a range of pro‐inflammatory cytokines expressed by osteocytes, with TNF‐α presenting as the candidate mostly likely to explain the patterns of decreased proliferation and increased migration resulting from osteocyte conditioned media. Thus, we identify a new mechanism through which invading cancer cells, initially entering an environment regulated by osteocytes, progressively corrupt this environment through disruption of osteocyte signaling. Indeed, this inhibition of cancer cell proliferation by osteocytes may play a role in the observed dormancy of breast and prostate cancer cells before establishing metastatic colonies in bone tissue.^[^ ^32^, ^33^, ^34^ ^]^

It is particularly interesting that this anti‐proliferative signaling can be shut down via soluble TGF‐β, which was secreted in large quantities by all four breast and prostate cancer cell lines. As mentioned above, osteocytes may encourage an apparently dormant state for metastatic cells via TNF‐α, a theory supported by trends of higher recurrence‐free survival in patients with higher TNF‐α receptor expression in the TCGA.^[^ ^27^ ^]^ In our working model of this mechanism the invading metastatic cancer cells produce TGF‐β, initially in small quantities, which begins to shut down this anti‐cancer mechanism upon contact with osteocyte TGF‐β receptors. This would be exacerbated via cancer cells upregulating osteoclast activity through other established pathways,^[^ ^35^, ^36^ ^]^ releasing TGF‐β sequestered in the surrounding bone matrix and further flooding the microenvironment with TGF‐β.^[^ ^37^ ^]^ Therefore, we speculate that a tipping point could be reached, with sufficient TGF‐β present to shut down the anti‐cancer TNF‐α mechanism locally, resulting in cancer cell proliferation, tumor growth and secretion of yet more TGF‐β, setting off a proliferative positive feedback loop. Elucidating this molecular mechanism presents the opportunity of inhibiting this feedback loop through disruption of the TGF‐β pathway. Indeed, it is well‐established that TGF‐β is crucial for development of bone metastases in vivo^[^ ^38^ ^]^ and small molecule TGF‐β inhibitors as adjuvant therapy have been shown to reduce EMT in a mouse model metastatic breast cancer^[^ ^39^ ^]^ with the prospect that further inhibition could shut down this mechanism. Alternatively, interventions to increase TNF‐α secretion by osteocytes or other cells in the bone environment could cause a similar therapeutic effect.

Osteocytes regulate bone homeostasis in response to mechanical loading via a range of mechanosensing mechanisms,^[^ ^40^, ^41^, ^42^ ^]^ including integrin attachments,^[^ ^43^, ^44^, ^45^ ^]^ their glycocalyx,^[^ ^46^, ^47^ ^]^ and the primary cilium.^[^ ^48^, ^49^ ^]^ Thus, changes in osteocyte behavior induced by TGF‐β from cancer cells may disturb bone biology, with disruptions to osteocyte regulation of bone remodeling linked to age‐related degeneration^[^ ^50^ ^]^ osteoporosis,^[^ ^51^, ^52^, ^53^ ^]^ osteoarthritis^[^ ^54^ ^]^ and osteogenesis imperfecta.^[^ ^55^ ^]^ In particular, it is well‐established that primary cilia are key to normal osteocyte function, mechanoresponsiveness and regulation of other bone cell types,^[^ ^56^ ^]^ and thus the cancer cell‐induced changes in osteocyte cilia we observed will likely also affect bone biology, such as the disruption of mechanosensitivity observed here. We have demonstrated here, for the first time, an additional role for osteocyte primary cilia and the associated IFT88 pathway, in controlling TNF‐α secretion and altering metastatic cancer cell behavior. Decreased expression and length of osteocyte cilia correlated with restoration of cancer cell proliferation. This presents the intriguing prospect that metastatic bone disease could in fact be treated as an osteocyte ciliopathy, with the potential to inhibit development and growth of metastatic lesions, and associated deterioration of bone tissue, via drugs that increase osteocyte primary cilia expression, such as fenoldopam.^[^ ^57^, ^58^, ^59^ ^]^ Further research is required to solidify this link, as the effect resulting from TGF‐β may be associative rather than causative, but these findings present a promising new therapeutic target for metastatic bone disease.

In conclusion, this study presents a novel anti‐cancer mechanism inherent in osteocytes, by far the most abundant bone cell type. We show that osteocytes suppress proliferative behavior in both breast and prostate cancer cells via secretion of soluble TNF‐α. However, this mechanism can be inhibited by cancer cells through secretion of TGF‐β, reducing osteocyte primary cilia and IFT88 expression, which downregulates TNF‐α secretion. These findings reveal a previously unknown mechanism regulates cancer cell proliferation and migration common to both breast and prostate bone metastases. This presents promising therapeutic targets, with the potential to reduce metastatic bone tumor growth in cancer patients.

## 4 Experimental Section

### Experimental Design

An array of cell culture experiments were designed in order to investigate the differences between early metastases, in which osteocyte signaling and regulation of the bone marrow environment likely dominates, and established metastases, in which the large numbers of cancer cells in a lesion likely results in crosstalk with osteocytes. Thus, conditioned media studies were used to mimic the one‐way signaling in early metastasis, while transwell co‐cultures were used to replicate the cytokine crosstalk via soluble factors in established metastases, as outlined in Figure 1A.

### Cell Culture Conditions

The human breast cancer cell lines MDA‐MB‐231 and MCF‐7, and human prostate cancer cell lines PC3 and LNCaP were obtained from the American Type Culture collection (ATCC), and were routinely maintained in Dulbecco's modified Eagle's medium (DMEM, Gibco) supplemented with 10% foetal bovine serum (FBS), and 100 U mL^−1^ penicillin and 100 µg mL^−1^ streptomycin (all Sigma–Aldrich). The MLO‐Y4 osteocyte‐like mouse cell line was a kind gift from Professor L. Bonewald (University of Missouri, Kansas City, USA) and were cultured on collagen‐coated surfaces (rat tail collagen type I, 0.15 mg mL^−1^) with α‐modified essential medium (α‐MEM, Gibco) supplemented with 2.5% FBS, 2.5% iron supplemented calf serum (CS, HyClone Laboratories, Logan, UT, USA), and 100 U mL^−1^ penicillin and 100 µg mL^−1^ streptomycin (all Sigma–Aldrich). hMSC's were routinely sub‐cultured in DMEM Glutamax (ThermoFisher 21885‐025) and supplemented with 10% FBS and 5% Penicillin/Streptomycin. Cells were differentiated at 5 × 10^3^ cell cm^−2^ in αMEM media supplemented with 10% FBS, 5% Penicillin/Streptomycin, 100 nM Dexamethasone, 5 µM Ascorbic Acid and 10 mM B‐glycerophosphate for a period of 21 days with media changed every 2–3 days. All cells were maintained at 37 ˚C, with 5% CO_2_ and 95% humidity.

In all cases, conditioned media (CM) from MLO‐Y4 cells was applied to cancer cells at a 1:1 ratio. Un‐cultured MLO‐Y4 standard media was applied at a 1:1 ratio to cancer cells in control groups, to remove any variability from combining different media types. The same technique was applied when adding cancer cell CM to osteocytes or hMSCs. Unless otherwise stated, each CM experiment contained three sample wells and was repeated on three separate occasions, resulting in *n* = 9 samples per group. In all co‐culture experiments, osteocytes were seeded at the same density as CM experiments and cultured for the same length of time, sharing the same total volume of media with the cancer cells.

### 3D Cell Cultures

MCF‐7 and PC3 cells were cultured as spheroids using the 3D on‐top assay as described in detail in Bissell et al.^[^ ^60^ ^]^ In brief, Matrigel was defrosted overnight and a thin layer of Matrigel was used to coat the cell culture plastic. Cells were seeded at .25 × 10^5^ cells cm^−2^ using 100 µL of Matrigel and incubated at 37 °C for 20 min. Cells were then overlaid with a 2% Matrigel supplemented media. Spheroids were cultured for a period of 12 days with a control or hMSC conditioned media (1:1 ratio) that was refreshed every 2–3 days. Spheroids were tracked and imaged using the Lumascope 720 and spheroid area was measured using the polygon tool in Fiji‐ImageJ.

### Organ‐on‐a‐Chip Model

The Emulate S1 chip was activated as per the standardized Emulate Inc protocol. Following the 21 days hMSC differentiation cells were trypsinized using 0.05% trypsin. Cancer spheroids were harvested from Matrigel after 12 days using a 5 mm EDTA‐PBS solution. The hMSC and cancer spheroids were then combined into a 1 mg mL^−1^ collagen‐I rat tail solution and seeded into the bottom channel of the Emulate S1 chip. The gel was allowed to set for 30 min before connecting the chips. The standard flow rate of 30 µL h^−1^ was applied to the top channel, while no flow was applied to the bottom channel.

### Fluid Shear Stress Experiments

Mechanical loading was applied to the MLO‐Y4 cells using oscillatory fluid flow generated by culturing cells in rectangular flasks (82 mm × 92 mm; 10 mL of media) on a rocking platform that oscillated at a frequency of 0.5 Hz and with an amplitude of 1.5 cm for 24 h after an initial 24 h static period post‐seeding. This system has been shown to generate spatiotemporal fluid‐flow induced maximal shear stress of ≈0.1 Pa across a layer of cells^[^ ^49^, ^61^ ^]^ that is partially representative of that experienced by osteocytes within the lacunar network in bone (0.01–1 Pa).^[^ ^42^, ^46^, ^62^, ^63^ ^]^ In all experiments, CM was collected after 24 h of fluid shear or un‐sheared static culture conditions.

### Proliferation Assay

The proliferation of the cultured cells was assessed using the AlamarBlue cell viability assay that detected redox reduction during cell growth. Cancer cells were seeded onto 24 well plates at a density of 25 × 10^3^ cells cm^‐^^2^ At the experimental endpoint, 50 µL of the AlamarBlue reagent (Life Technologies, Eugene, OR, USA) was added to each well containing cells and 500 µL of culture medium. Cells were then incubated for 3 h at 37 ˚C. The fluorescence was measured with a Synergy 4 multi‐mode microplate reader (BioTek Instruments, Winooski, VT, USA) with excitation at 544 nm and emission at 590 nm. The fluorescence value was proportional to the number of viable cells.

### Migration Assay

Cancer cells were seeded into a 24‐well plate at a density of 50 × 10^3^ cells cm^−2^ and allowed to form a monolayer that was then scratched with a P200 pipette tip to create a linear wound ≈200 µm wide. Migration of the cells into the wounding gap was monitored by light microscopy serial time‐lapse imaging using a Lumascope LS720 imaging system (Etaluma Inc., Carlsbad, CA, USA) with a 20× objective. The percentage of wound gap closure was measured using a plugin for ImageJ software (National Institutes of Health, Bethesda, MD, USA) as previously described.^[^ ^64^ ^]^

### Invasion Assay

An in vitro Matrigel invasion assay was used to assess the invasiveness of cancer cells^[^ ^65^ ^]^ Briefly, transwell inserts (8‐µm pores) for 24‐well plates were pre‐coated with 50 µL/insert of 1 mg mL^−1^ Matrigel (Corning Inc., Corning, NY, USA), for 1 h at 37 ˚C. Subsequently, cancer cells were seeded into the upper chamber of each insert at 75 × 10^3^ cells cm^−2^ in 250 µL basal medium. Control medium or CM, 500 µL, was added to each well (lower chamber) under the inserts. After incubation for 24 h, cells that had penetrated the Matrigel‐coated membrane and adhered to the other side of the inserts were dissociated with Trypsin (Sigma–Aldrich) for 7 min at 37 ˚C. A total of 250 mL of media was then added to neutralize the Trypsin. AlamarBlue was then added to the solution containing invaded cells, with the assay performed as described for proliferation above.

### Enzyme‐Linked Immunosorbent Assay (ELISA)

TGF‐β secretion by cancer cells, and TNF‐α secretion by osteocytes, was quantified via enzyme‐linked immunosorbent assay (ELISA), according to manufacturer's instructions (Catalogue numbers 501 129 049 and BMS607‐3, respectively; both Invitrogen Life Technologies, Eugene, OR, USA). Control and conditioned media were added to a coated Corning ELISA plate. Samples were washed three times and incubated with horseradish peroxidase‐conjugated secondary antibody for 1 h at room temperature. Horseradish peroxidase detection reagent was added, the samples were incubated at room temperature for 30 min, and absorbance was measured at 450 nm.

### Immunocytochemistry and Microscopy

For primary cilia imaging and analysis, MLO‐Y4 cells cultured on collagen I‐coated glass‐bottom 24‐well plates (Mattek) were fixed in 10% formalin and treated with anti‐acetylated α‐tubulin primary antibody, 1:1, from a C3B9 hybridoma cell line (Sigma–Aldrich). Cilia were visualized with Alexa‐Fluor 488 secondary antibody, 1:1000 (Life Technologies) and imaged with a 100× oil objective on a Leica DMi8 epifluorescence microscope. Nuclei were stained with DAPI (Life Technologies), and F‐actin was stained with phalloidin (Santa Cruz Biotech). Cell area, and circularity, and cilia incidence and length were analyzed using ImageJ software.

### Confocal Microscopy

Imaging of the organ‐chip was performed at 20× on a Zeiss 710 ELYRA PS.1 confocal microscope using an EC PlanNeofluar10×/0.3 M27 objective (Zeiss, Oberkochen, Germany). Confocal z‐sections were made throughout the cell depth (approximately 20 sections) using 5 µm step size with an image format of 2048 × 2048 yielding a pixel size of 0.415 µm × 0.415 µm (image size ≈850 µm × 850 µm).

### Super‐Resolution Structured Illumination Microscopy

Cells imaged for super‐resolution structured illumination microscopy were cultured on coverslips, fixed with 10% formalin, and underwent permeabilization with both 0.5% Triton X‐100 and Methanol. The ciliary axoneme was detected using a mouse acetylated α‐tubulin antibody (1:2000, Sigma–Aldrich). The basal body was observed using rabbit pericentrin (1:500, Abcam), and the intraflagellar transport protein, homolog 88, was detected using an IFT88 polyclonal rabbit antibody (1:1000, Proteintech). Slides were mounted using ProLong Antifade mountant (Invitrogen) and imaged using the Zeiss 710 ELYRA PS.1 microscope (Carl Zeiss, Oberkochen, Germany) with a 63×/1.4 NA objective.

### Cytokine Array

Cytokines present in osteocyte CM were assessed using an Abcam mouse cytokine antibody array (ab133994, Abcam) according to manufacturer's instructions. Image Studio Lite was used to quantitate cytokine spots as per manufacturer's instructions, measured using Li‐Cor Odyssey imaging system. Raw densitometry data was extracted by identifying a single exposure with a high signal to noise ratio, measuring the density of each spot using circles of equal size dimensions, and determining the summed signal density across the entire circle for each spot. Background signal was then subtracted and data was normalized based on positive control signals for each array. A negative control of uncultured standard media and a positive control of osteocytes treated with lipopolysaccharide (LPS), known to stimulate an inflammatory response in osteocytes^[^ ^66^ ^]^ were included.

### Western Blotting

Cells were lysed in RIPA lysis buffer (Sigma) with complete protease inhibitor cocktail (Sigma) and PhosSTOP (Sigma). Lysates were denatured by boiling for 5 min with SDS loading buffer, separated on a 14‐10% Bis Tris gel (Invitrogen) and transferred to polyvinylidene difluoride (PVDF) membrane. Membranes were activated using 100% methanol prior to transfer and were subsequently blocked using 5% BSA at room temperature for 1 h. Primary antibodies were used at 4 °C overnight at a 1:1000 dilution (TGF‐β Receptor 1: Abcam, IFT88: Proteintech, β‐Actin: Abcam). Following incubation and membrane washing with 1x TBS‐T, secondary HRP conjugated antibodies were used at room temperature for 1 h. Protein bands were detected using an enhanced chemiluminescence substrate enhancer solution that was applied to the membrane directly before scanning using either the GE healthcare chemidoc system or the Li‐Cor Odyssey imaging system.

### Cell Treatments

Cell lines were treated with the following where relevant prior to downstream assays: 10 µg mL^−1^ TGF‐β Receptor 1 small molecule inhibitor (LY 364 947, Tocris), 5 ng mL^−1^ recombinant human TGF‐β1 (240‐B‐002, R&D Systems), 10 ng mL^−1^ Lipopolysaccharide (LPS) solution (00‐4976‐93, Invitrogen), 10 ng mL^−1^ recombinant human TNF‐α (PHC3015, Gibco), 10 ng mL^−1^ recombinant human IL‐6 (PHC0064, Gibco), 10 ng mL^−1^ recombinant human IL‐10 (PHC0104, Gibco), 10 ng mL^−1^ recombinant human VEGF‐A (PHC9394, Gibco), 1 ng mL^−1^ TNF‐α Receptor small molecule inhibitor (CAS 1049741‐03‐8, Calbiochem, Sigma–Aldrich).

### RNA Interference

Gene silencing was performed by siRNA mediated knockdown and compared to scramble siRNA control (Life Technologies). For TGF‐β receptor 1 disruption, cells were transfected with 20 µM TGF‐βR1 siRNA (5′‐CGAACAGAAGUUAAGGCCAAAUAUU‐3′). For primary cilia disruption, cells were transfected with 20 µM IFT88 siRNA (5′‐CCAGAAACAGATGAGGACGACCTTT‐3′) or scrambled siRNA control using Lipofectamine 2000 (Life Technologies) as previously described^[^ ^67^ ^]^ Any gross effects were not observed on cellular morphology for all siRNA treatments.

### RNA Extraction

RNA was extracted from cells using the RNeasy Plus Minikit (Qiagen) as per manufacturer instructions. RNA was quantified using a Nanodrop Spectrophotometer (Thermo scientific) and all RNA was stored at −80 °C. Using the Quantitech reverse transcription kit (Qiagen) 1 µg of total RNA was synthesised into cDNA and stored at −20 °C.

### RT‐qPCR

Real time PCR was performed using TaqMan gene expression assay kits (Thermo Fisher) and the Quant Studio 7 flex real time PCR system (Thermo Fisher). Gene expression was analyzed by quantitative real‐time PCR using primers and probes (Life Technologies) for analysis of intraflagellar transport 88, *IFT88* (Mm00493675_m1); cyclooxygenase‐2, *COX‐2* (Mm00478374_m1) and *GAPDH* (4 351 309). *GAPDH* was used as a housekeeping gene endogenous control and relative fold change in gene expression was calculated using the 2^−ΔΔCT^ method.

### Gene Expression and Protein Interaction Database Analyses

A protein‐protein interaction (PPI) network was generated from experiments and datasets only, using the high confidence setting on STRING (Search Tool for the Retrieval of Interacting Genes/Proteins).^[^ ^26^ ^]^ Clustering analysis was performed using the Markov Cluster Algorithm (MCL) with the highest inflation parameter (10), indicating a cluster of highly interacting proteins among cytokine array targets.

Kaplan–Meir plots of recurrence‐free survival in patients with high or low expression of the TNFRSF1A gene were generated from The Cancer Genome Atlas (TCGA) using KMPlotter.^[^ ^27^ ^]^ Analysis was performed on the Pan‐Cancer DNA repositories, using n = 980 breast cancer and *n* = 492 prostate cancer samples.

A comparison of RNA expression of the TNFRSF1A gene between normal, primary tumor and mestastatic tumor samples was performed on databases of tumor samples using the TNMPlot tool^[^ ^28^ ^]^ that compared data from the Gene Expression Omnibus of the National Centre for Biotechnology Information (NCBI‐GEO), The Cancer Genome Atlas (TCGA), the Therapeutically Applicable Research to Generate Effective Treatments (TARGET), and the Genotype‐Tissue Expression (GTEx).

### Statistical Analysis

As described in figure legends, the statistical analyses were performed using GraphPad Prism 5 (GraphPad Software). Statistical significance compared between groups indicated by horizontal lines as follows: light gray, *p* < 0.05; dark gray, *p* < 0.01; black, *p* < 0.001; by one‐way ANOVA with Bonferroni post‐hoc test. As indicated in the figure legends, experiments were repeated independently multiple times and similar results were obtained.

## Conflict of Interest

The authors declare no conflict of interest.

## Author Contributions

S.W.V., C.R.J., and M.M.K. performed conceptualization. S.W.V., J.N., M.P.D., and M.M.K. performed methodology. S.W.V., J.N., and M.P.D. performed the investigation. S.W.V. and J.N. performed visualization. O.M.T.P., C.R.J., and M.M.K. performed supervision. S.W.V. wrote the original draft. S.W.V., J.N., M.P.D., O.M.P.T., and M.M.K. performed review and editing.

## Supporting information

Supporting Information

Supporting Information

## Data Availability

The data that support the findings of this study are available in the supplementary material of this article.
