# Supplementary material for: A Novel Primary Cilium‐Mediated Mechanism Through which Osteocytes Regulate Metastatic Behavior of Both Breast and Prostate Cancer Cells
Source: Adv Sci (Weinh). 2023 Nov 15;11(2):2305842. doi: 10.1002/advs.202305842 (PMC10787058; doi:10.1002/advs.202305842)
Supplement: Supplementary file 1 — Supporting Information [file ADVS-11-2305842-s002.pdf]

ADVANCED SCIENCE
Open Access

Supporting Information

for *Adv. Sci.*, DOI 10.1002/advs.202305842

A Novel Primary Cilium-Mediated Mechanism Through which Osteocytes Regulate Metastatic Behavior of Both Breast and Prostate Cancer Cells

Stefaan W. Verbruggen, Joanne Nolan, Michael P. Duffy, Oliver M.T. Pearce, Christopher R. Jacobs and Martin M. Knight*

A novel primary cilium-mediated mechanism through which osteocytes regulate metastatic behaviour of both breast and prostate cancer cells

Stefaan W. Verbruggen*, Joanne Nolan, Michael P. Duffy, Oliver M.T. Pearce, Christopher R. Jacobs, Martin M. Knight

*Corresponding author. Email: s.verbruggen@qmul.ac.uk

Supplementary Information

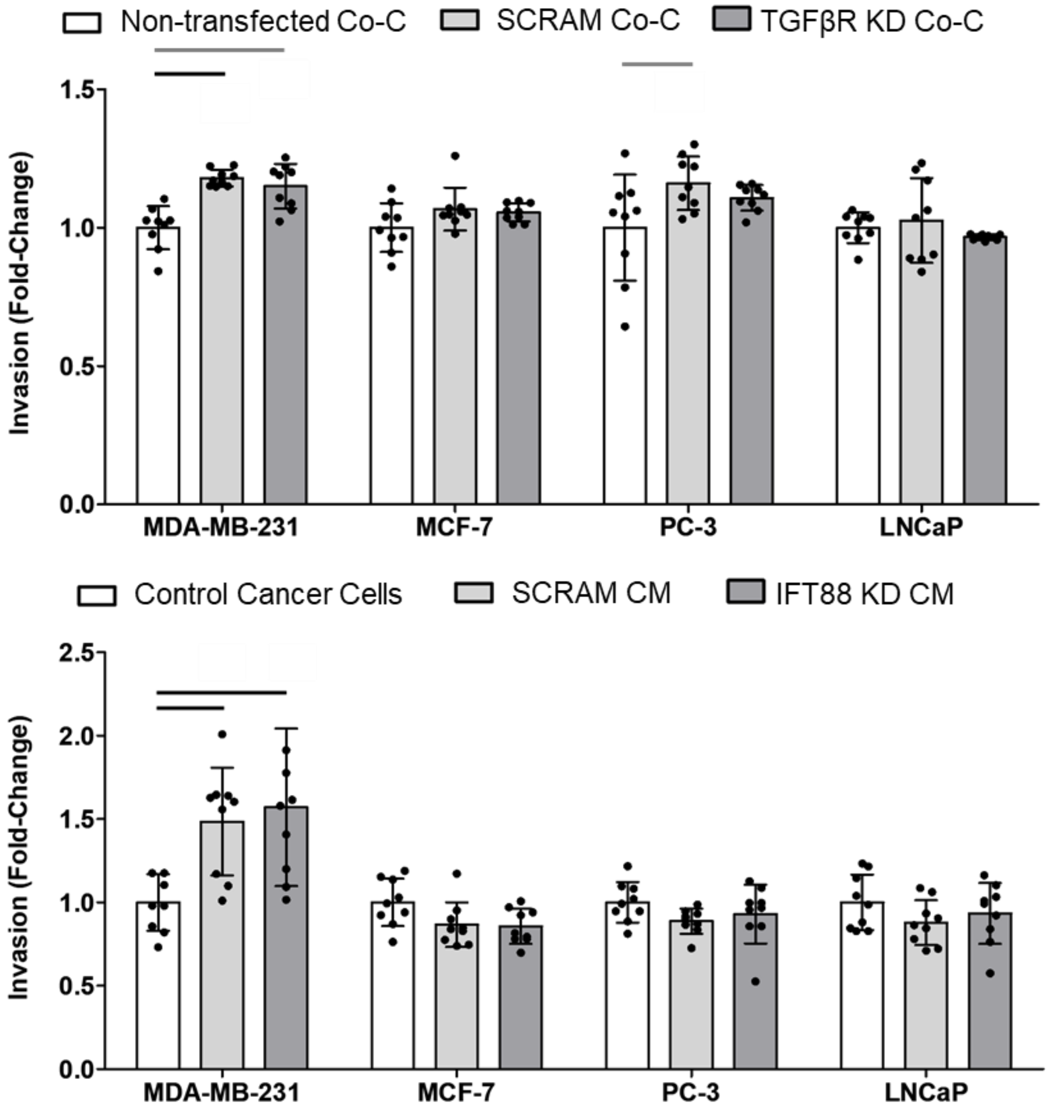

Fig.S1: Additional invasion data from subsequent co-culture (Co-C) and conditioned media (CM) experiments. Bar charts represent mean ± standard deviation. Statistically significant differences indicated by horizontal lines based on one-way ANOVA with Bonferroni post-hoc test (light grey $P<0.05$, dark grey $P<0.01$, black $P<0.001$).

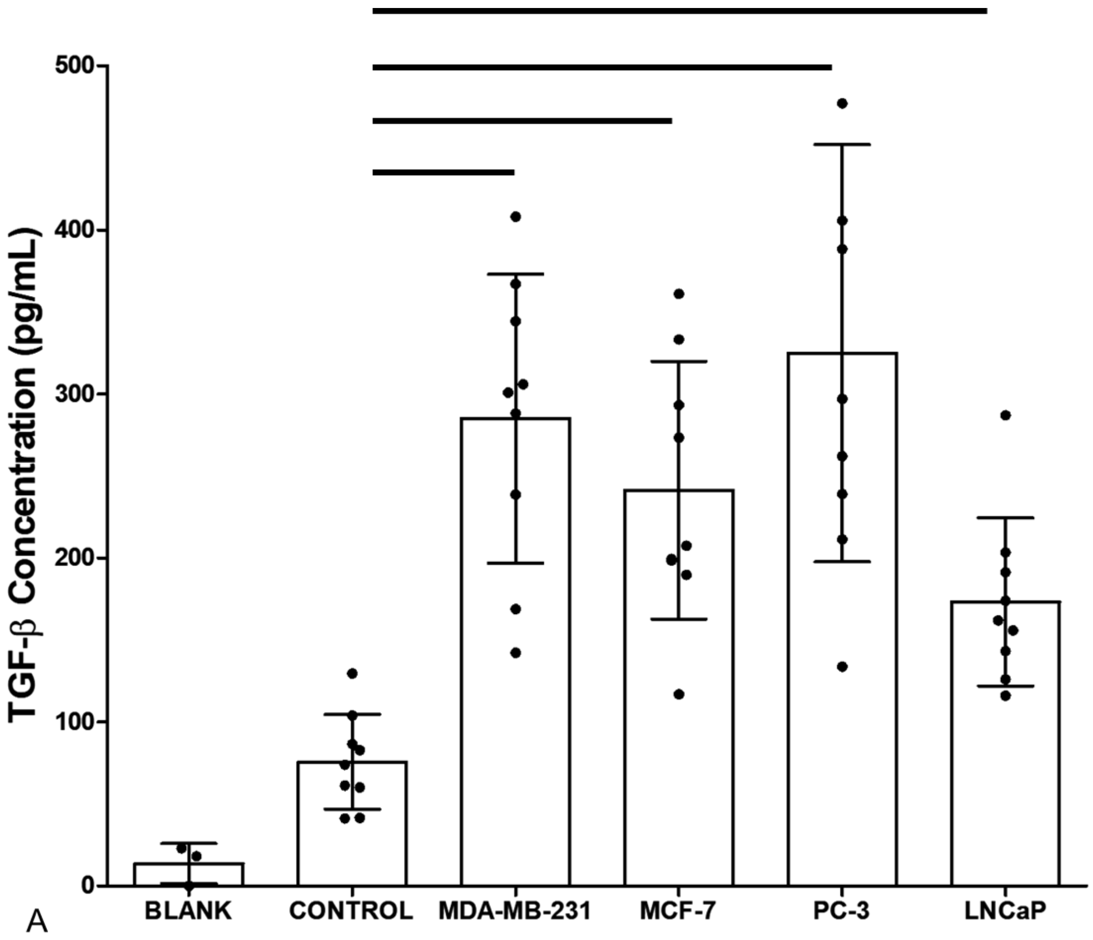

Fig.S2: Concentrations of TGF-β, as measured by ELISA. Bar charts represent mean ± standard deviation. Statistically significant differences indicated by horizontal lines based on one-way ANOVA with Bonferroni post-hoc test (light grey P<0.05, dark grey P<0.01, black P<0.001).

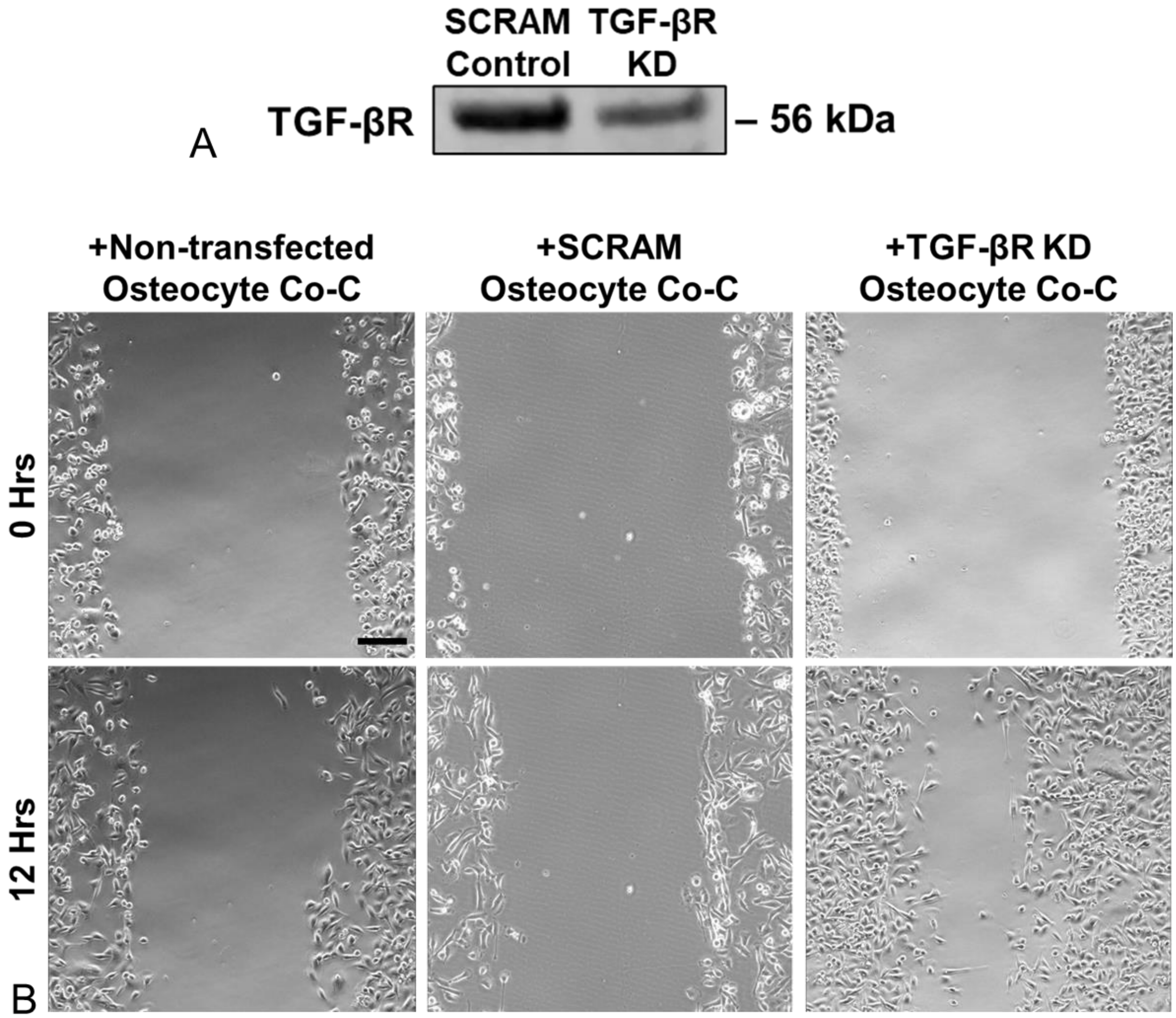

Fig.S3: Knockdown of osteocyte TGF-β Receptor 1 via siRNA transfection increases cancer cell proliferation in co-culture. (A) Western blot showing TGF-β Receptor 1 protein production by osteocytes and (B) representative images of scratch assay, showing the effect of osteocyte TGF-β Receptor 1 knockdown on MDA-MB-231 migration. Gap closure in scratch assay in co-culture imaged over 12 hours using Lumascope LS720. Scale bar = 100 µm

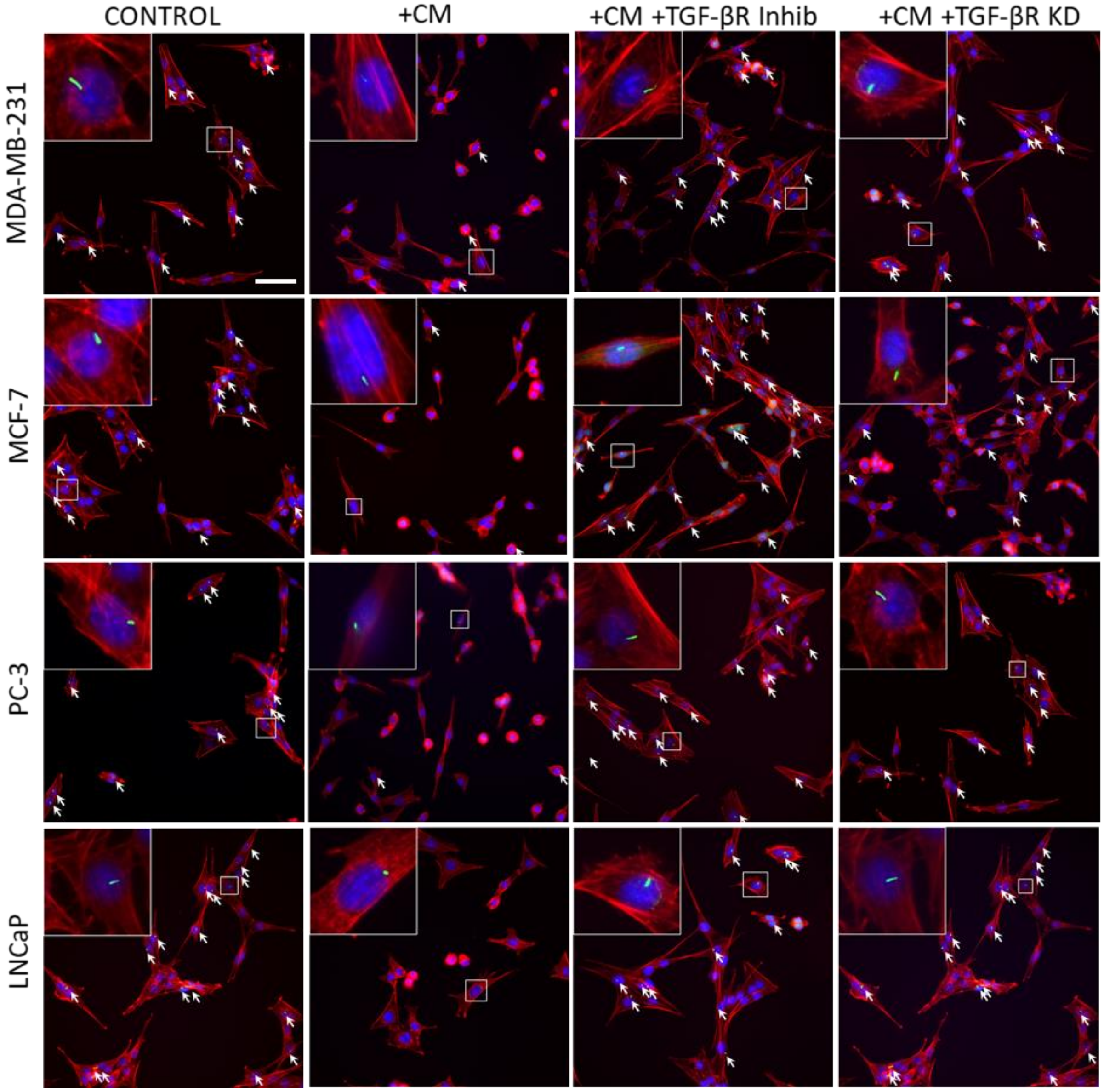

Fig.S4: Immunofluorescent images of osteocytes labelled for primary cilia (acetylated α-tubulin, green, arrows) and counter stained with phalloidin for F-actin (red) and DAPI for nuclei (blue). Significant reduction in primary cilia incidence and length, as well altered cell circularity and area, observed with conditioned media from both breast and prostate cancer cells, via TGF-β. Scale bar = 20 µm

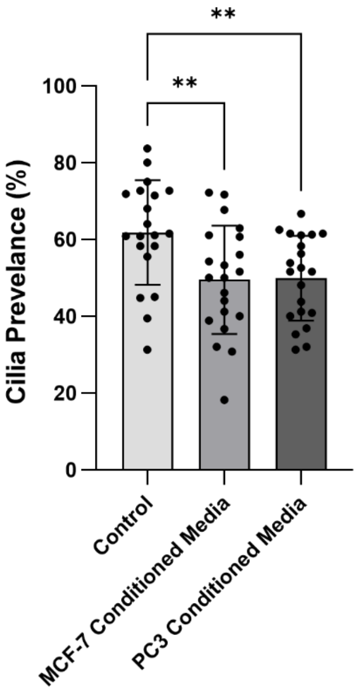

Fig.S5: Conditioned media from cancer cells reduced primary cilium expression in osteogenically-differentiated human MSCs in a similar manner to the MLO-Y4 mouse osteocyte cell line Quantification of primary cilia incidence osteogenically differentiated hMSCs, as measured by immunofluorescent labelling acetylated α-tubulin and DAPI. Bar charts represent mean ± standard deviation for n=9 technical replicates. Statistically significant differences indicated by horizontal lines based on one-way ANOVA with Bonferroni post-hoc test (** = P<0.01).

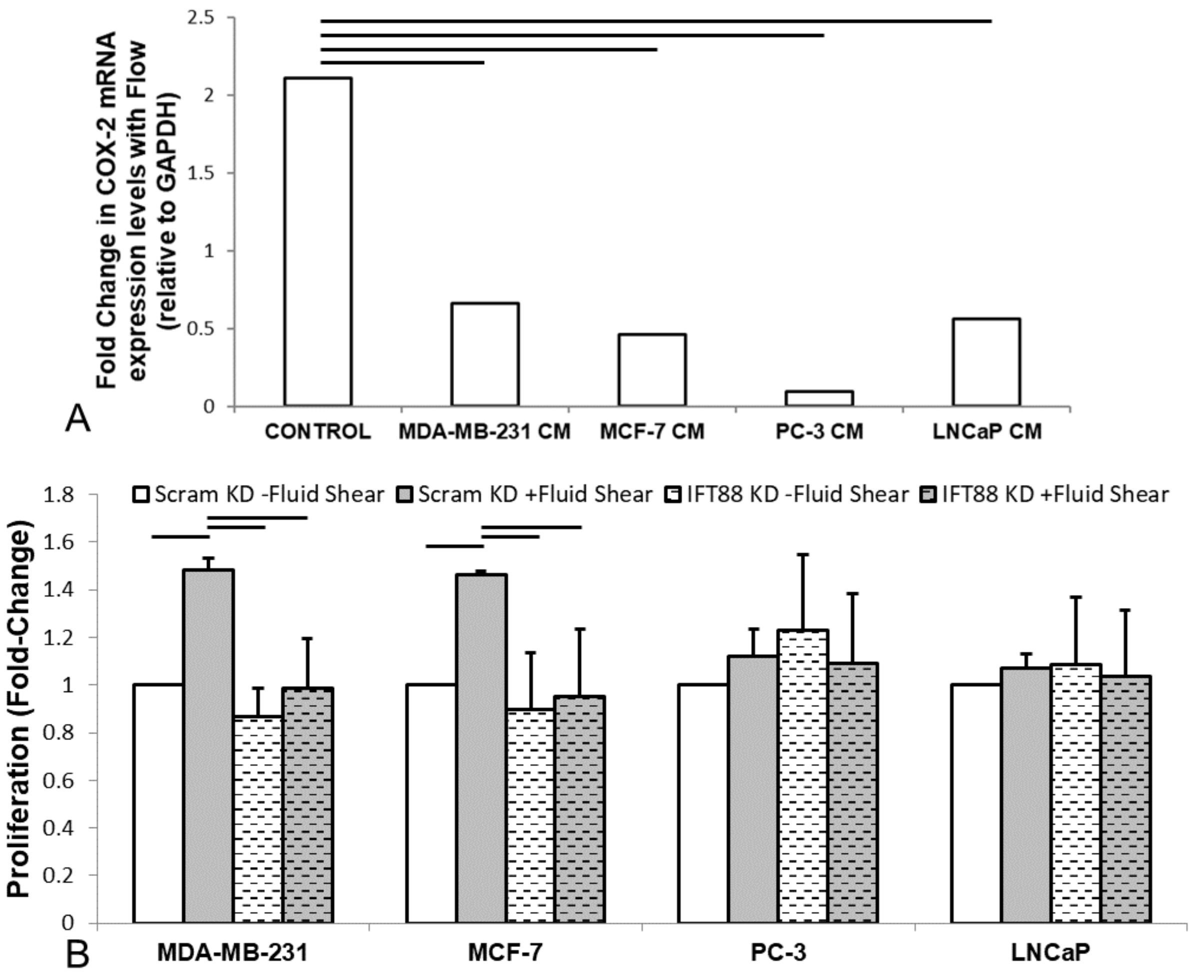

Fig.S6: Cancer cells decrease osteocyte mechanosensitivity, with downstream effects on breast, but not prostate, cancer cell proliferation. (A) Quantification of the effect of cancer cell conditioned media on MLO-Y4 osteocyte mechanosensitivity to flow, measured as COX-2 mRNA expression via qPCR relative to GAPDH and to static controls. (B) Artificial knockdown of osteocyte primary cilia abrogates their ability to regulate breast cancer cell proliferation via conditioned media, though it appears to have no effect on prostate cancer cells. Bar charts represent mean ± standard deviation for n=9 technical replicates. Statistically significant differences indicated by horizontal lines based on one-way ANOVA with Bonferroni post-hoc test ($P<0.001$).

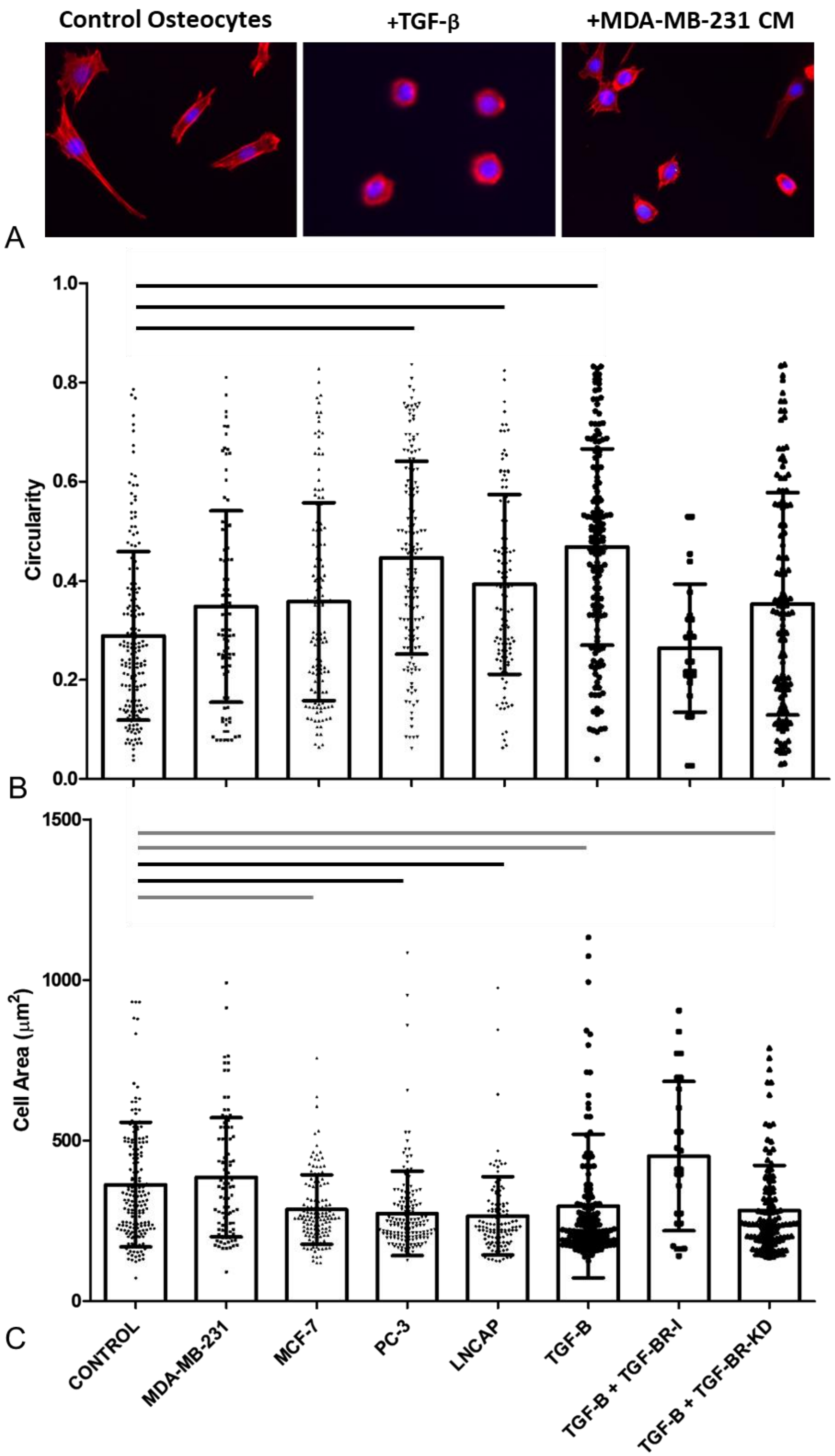

Fig.S7: TGF-β and cancer cell conditioned media treatment cause rounding of cell shape. (A) Immunofluorescent images of osteocytes labelled phalloidin for F-actin (red) and DAPI for nuclei (blue). Corresponding quantification of (B) circularity and (C) cell area. Bar charts

represent mean ± standard deviation for n=~200 cells over n=9 technical replicates. Statistically significant differences indicated by horizontal lines based on one-way ANOVA with Bonferroni post-hoc test (light grey $P<0.05$, dark grey $P<0.01$, black $P<0.001$).

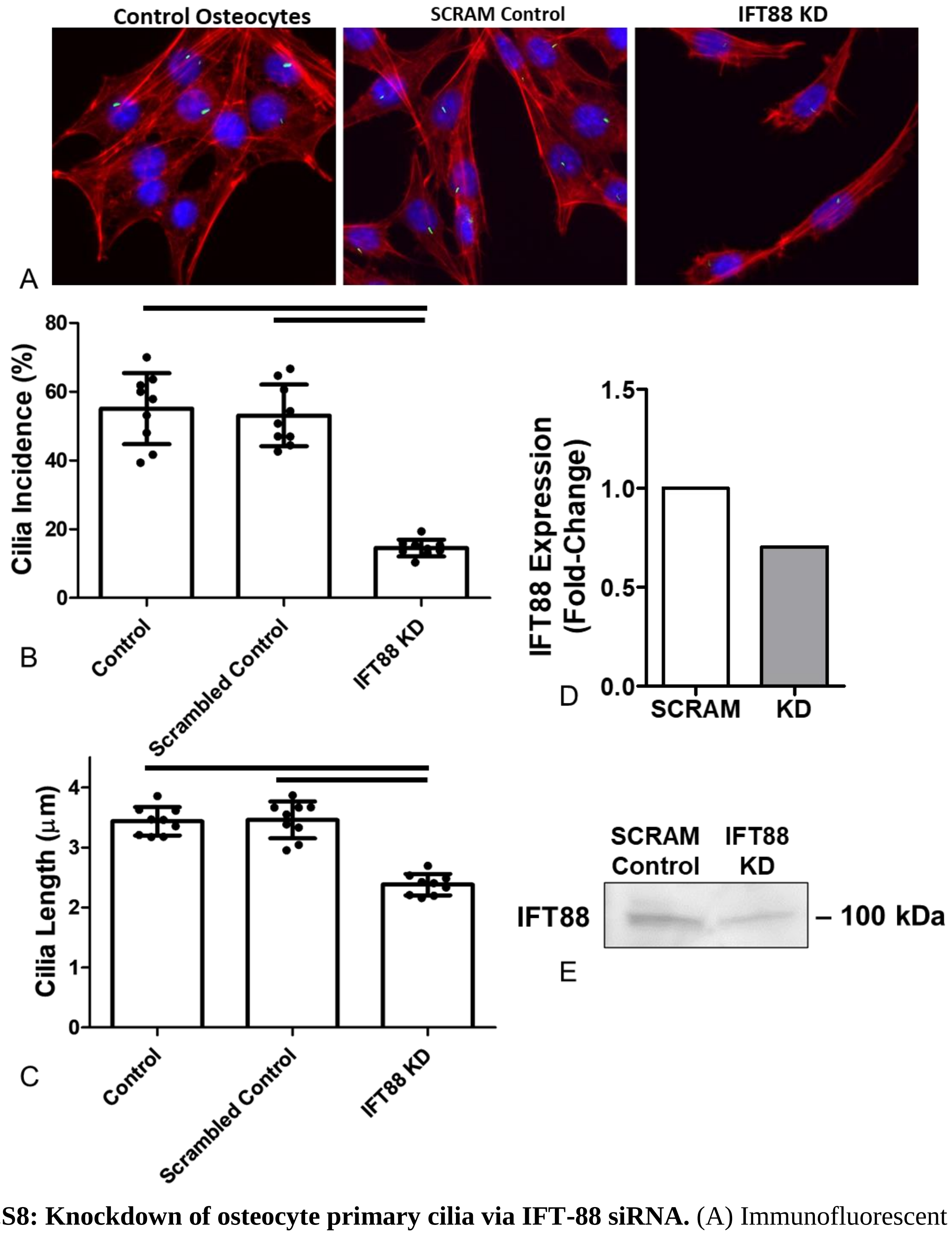

Fig.S8: Knockdown of osteocyte primary cilia via IFT-88 siRNA. (A) Immunofluorescent images of osteocytes labelled for primary cilia (acetylated α-tubulin, green) and counter stained with phalloidin for F-actin (red) and DAPI for nuclei (blue). Corresponding quantification of (B) cilia incidence and (C) cilia length. Confirmation of knockdown via (D) qPCR and (E) Western Blot. Bar charts represent mean ± standard deviation for n=9 technical replicates. Statistically significant differences indicated by horizontal lines based on one-way ANOVA with Bonferroni post-hoc test (light grey $P<0.05$, dark grey $P<0.01$, black $P<0.001$).

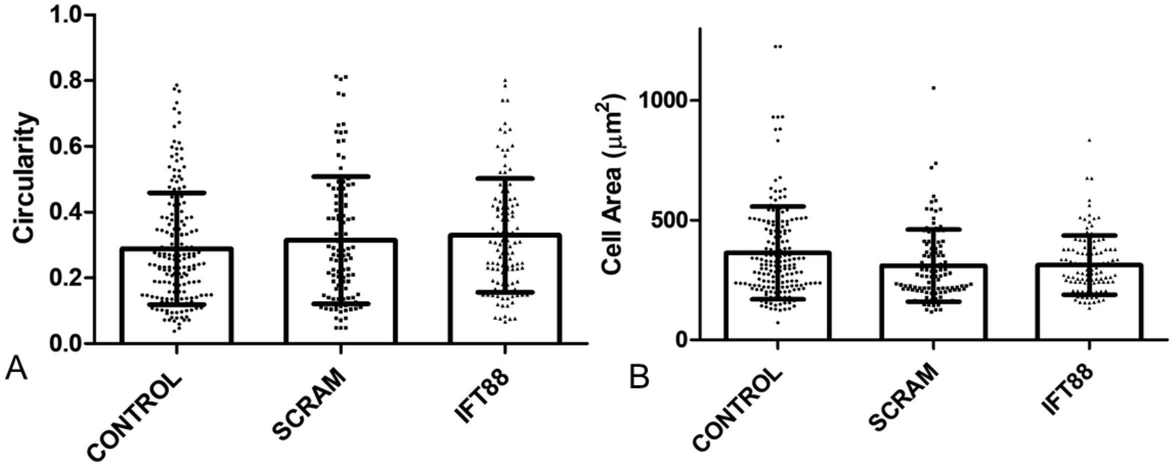

Fig.S9: IFT88 knockdown does not affect osteocyte cell shape. Osteocyte cell shape as measured by (A) circularity and (B) cell area. Bar charts represent mean ± standard deviation for n=~200 cells over n=9 technical replicates

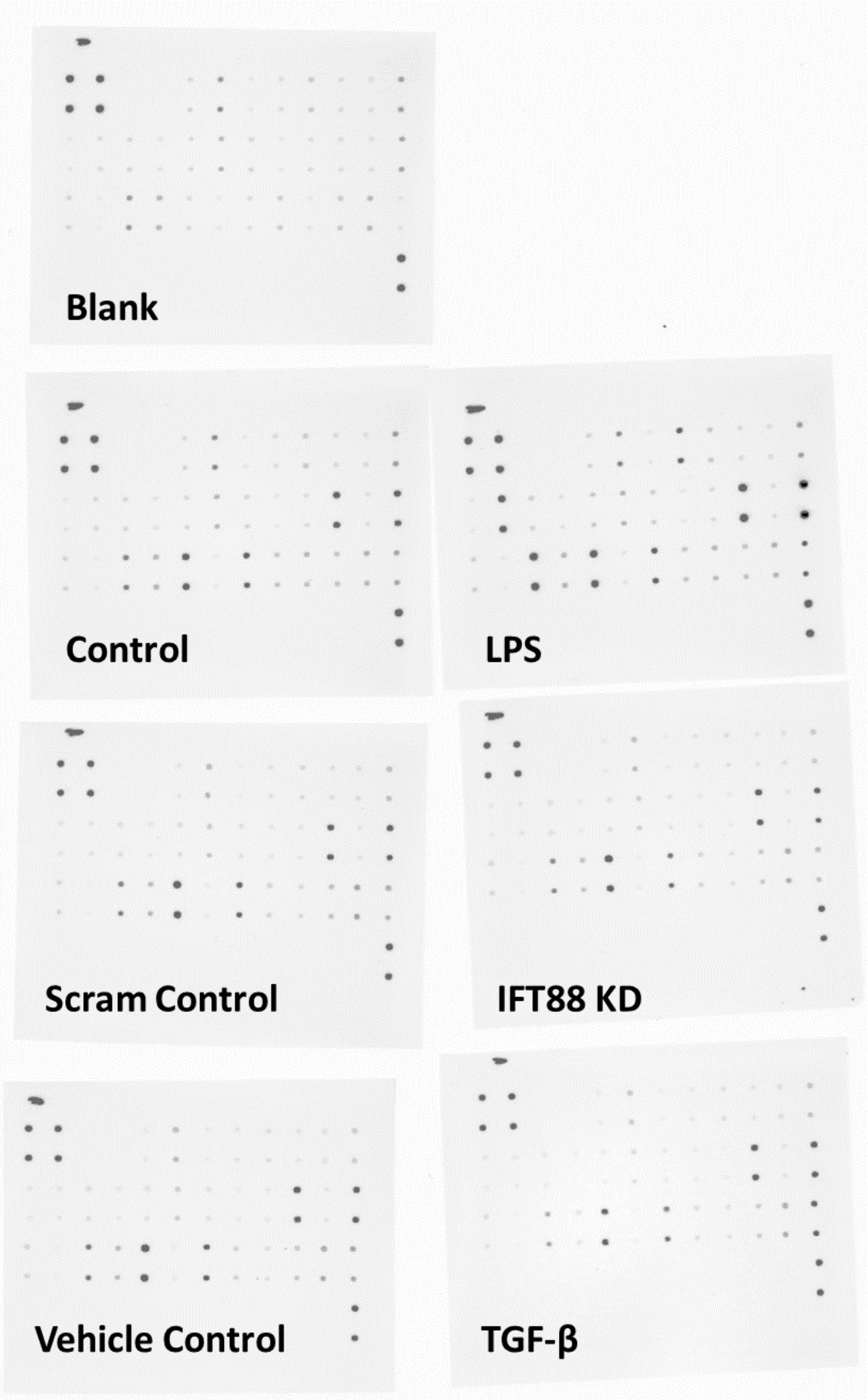

Fig.S10: Complete scan of cytokine array membranes. Conditions tested include blank standard cell culture media as negative control, control media from osteocytes in standard culture, media from osteocytes stimulated by pro-inflammatory lipopolysaccharides (LPS) as a positive control, as well as IFT88 knockdown and its Scrambles siRNA control, and TGF-β alongside its vehicle control.

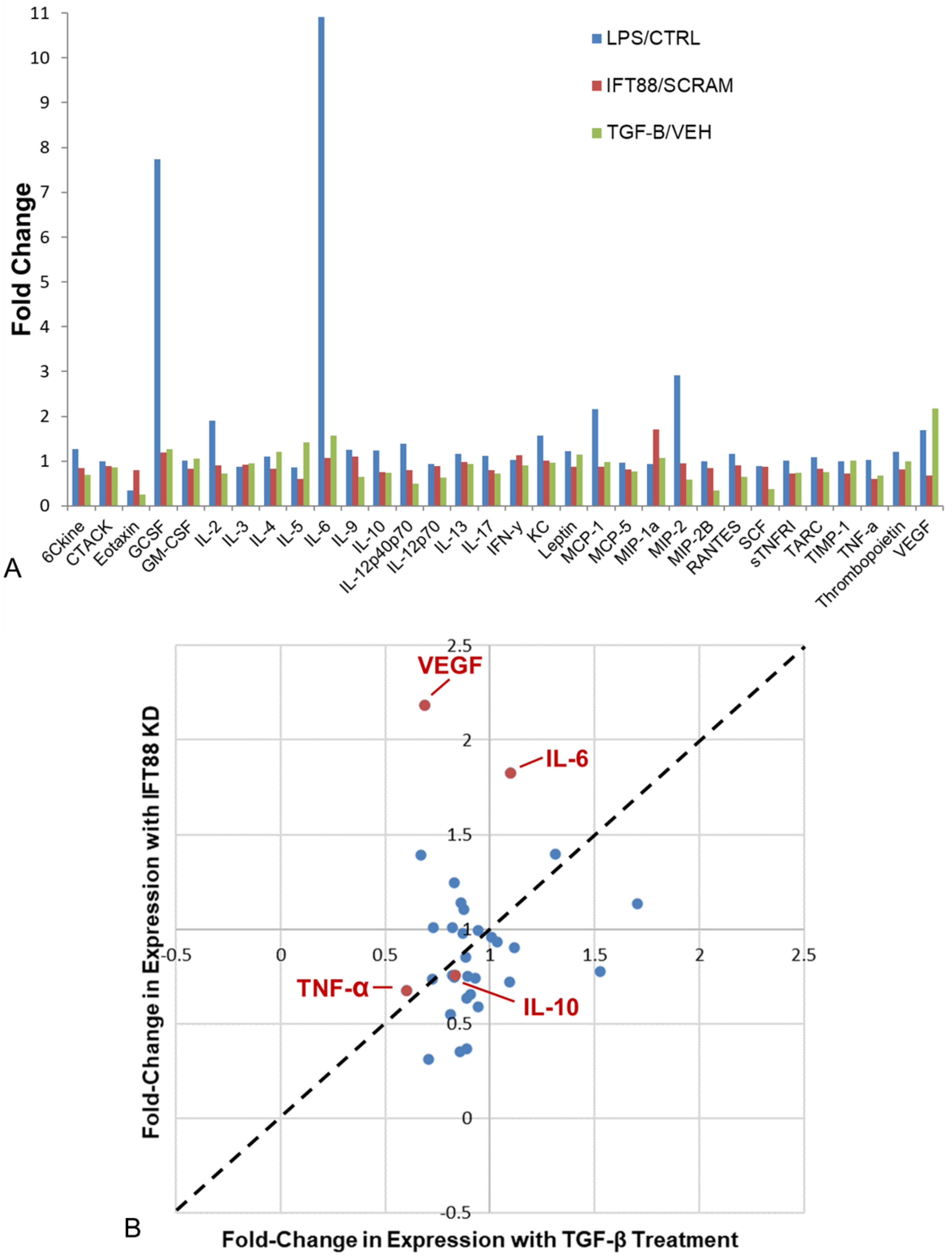

Fig.S11: Analysis of fold-changes in cytokine expression between LPS, IFT-88 siRNA or TGF-β treatment, and their respective controls. (A) Pro-inflammatory stimulation observed with LPS treatment as a positive control, evidence by upregulation of cytokine secretion. (B) 32 cytokines measured were compared for similarity of expression between IFT-88 siRNA and TGF-β treatment, with the dashed line representing 1:1 relationship, and the red indicating the cytokines selected for further analysis.

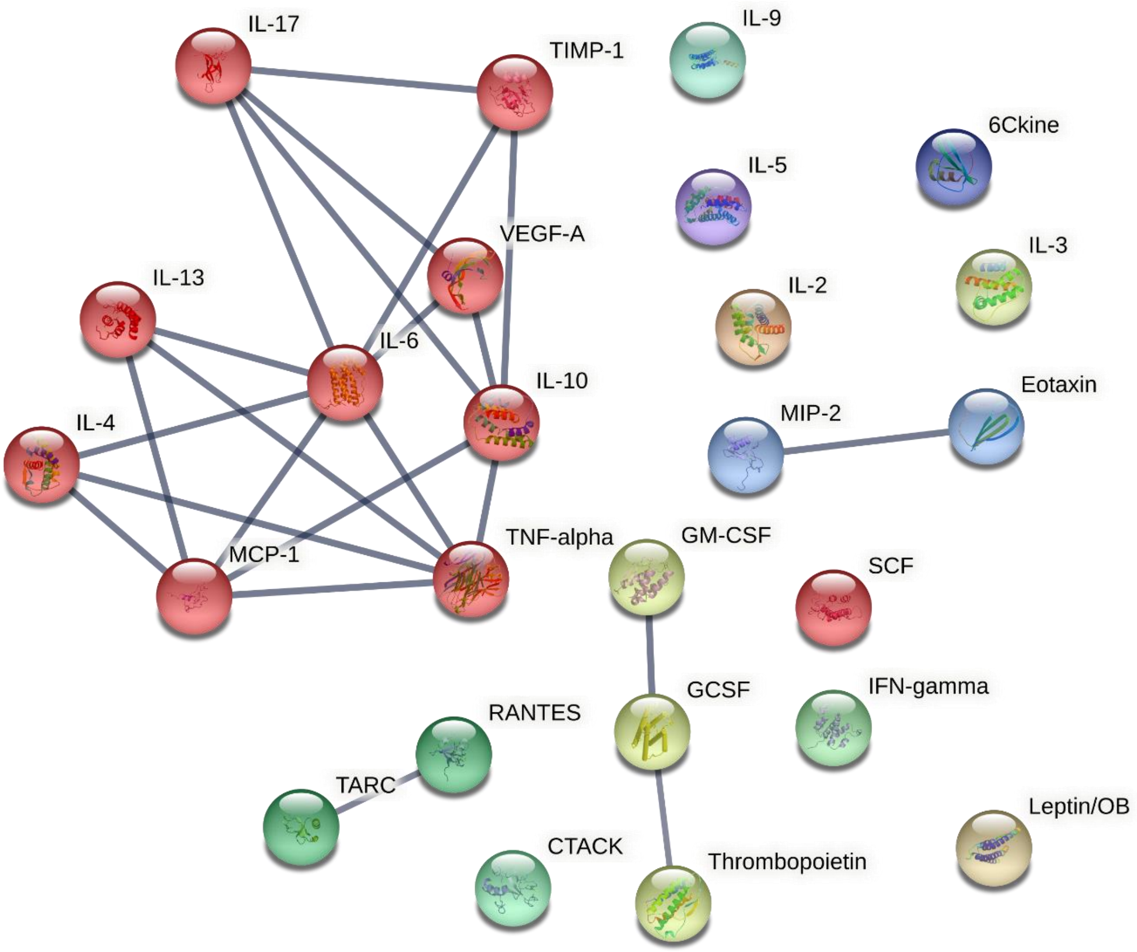

Fig.S12: Protein-protein interaction (PPI) network indicating a cluster of highly interacting proteins (red) among cytokine array targets generated using STRING software. Network generated from experiments and datasets only, using the high confidence setting on STRING (Search Tool for the Retrieval of Interacting Genes/Proteins). Clusters were obtained using the Markov Cluster Algorithm (MCL) with the highest inflation parameter (10).

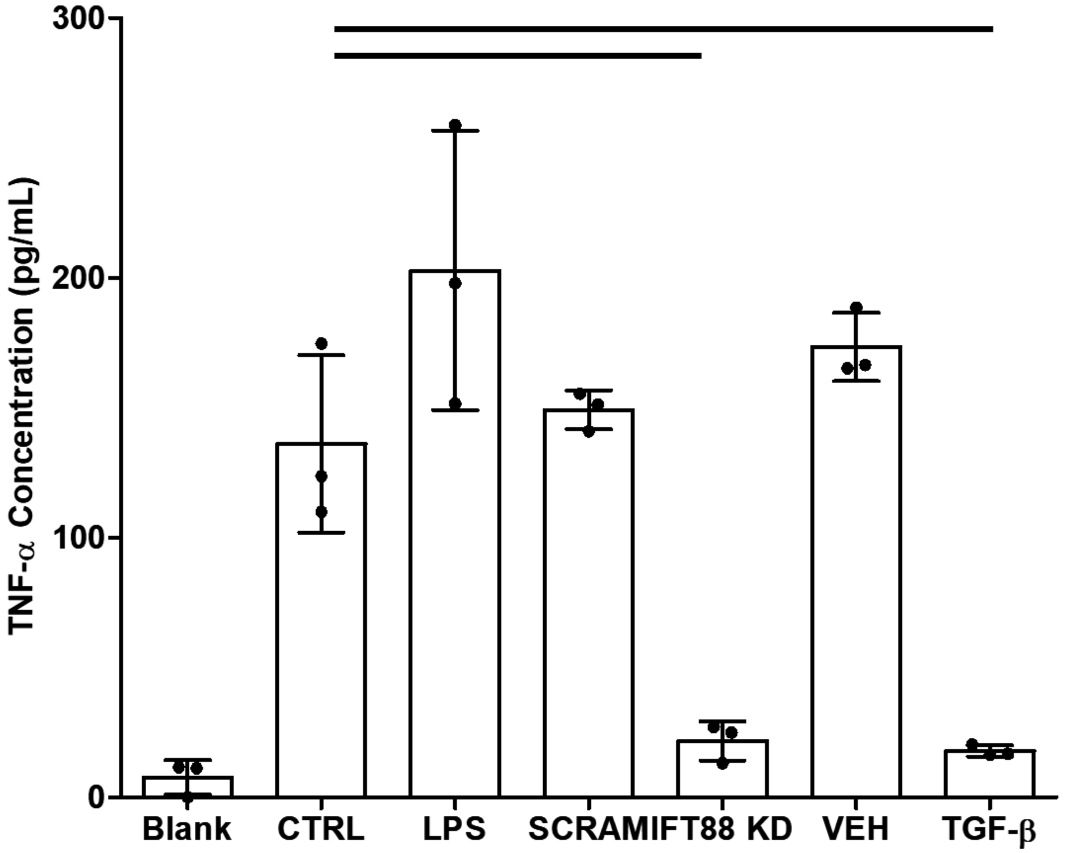

Fig.S13: Concentrations of TNF-α, as measured by ELISA. Bar charts represent mean ± standard deviation. Statistically significant differences indicated by horizontal lines based on one-way ANOVA with Bonferroni post-hoc test (light grey $P<0.05$, dark grey $P<0.01$, black $P<0.001$).

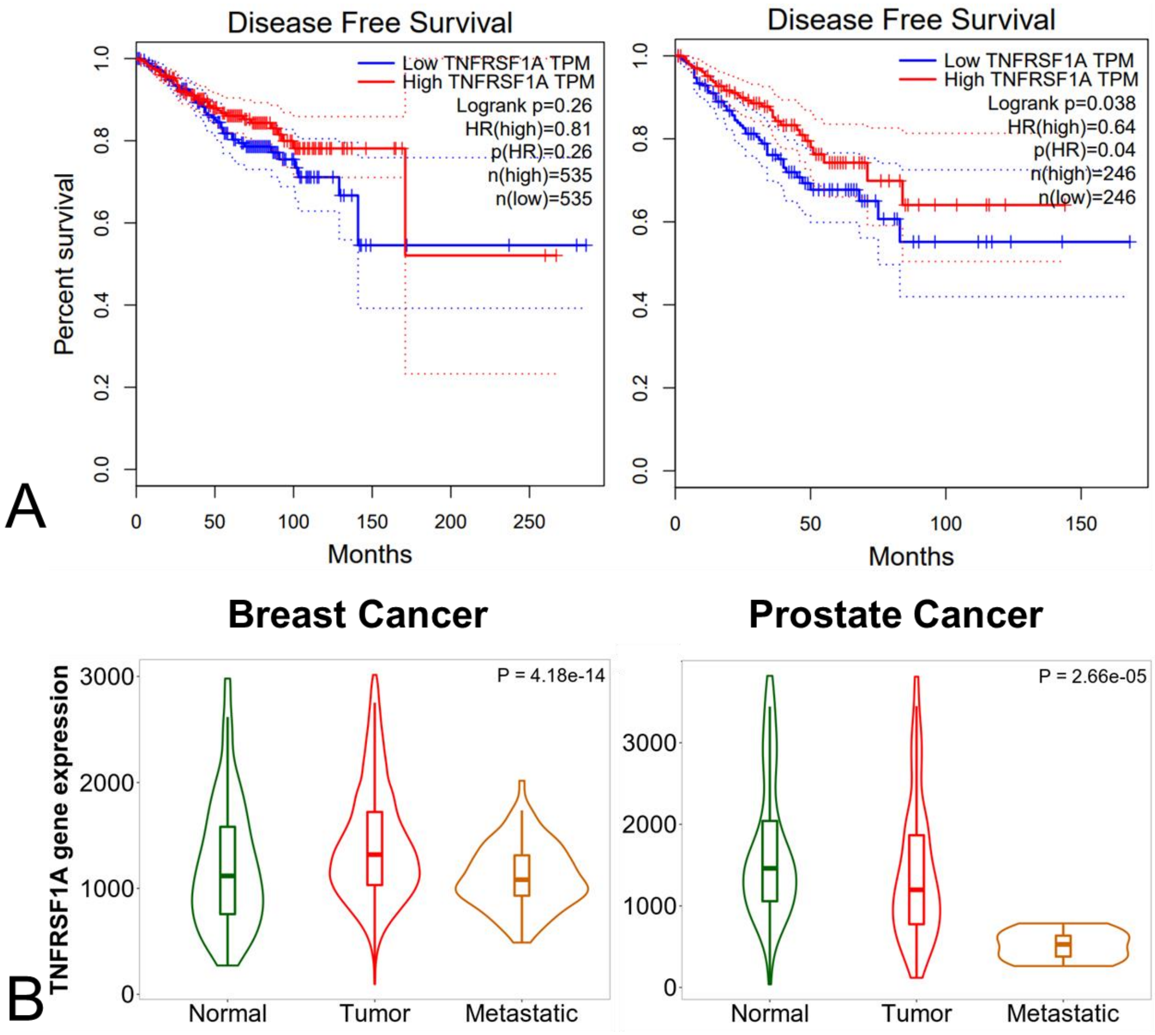

Fig.S14: Trends of low expression of genes encoding for TNF-α receptors in patients with lower disease free-survival and in metastatic tissue. (A) Kaplan-Meir plots of recurrence-free survival in patients with high or low expression of the TNFRSF1A gene, generated from TCGA using KMPlot. (B) Violin plots showing significant decreases in expression of the TNFRSF1A gene in metastatic tissue samples, compared to Normal or Primary Tumour samples, generated from databases using TNMPlot.
